# High-Yield α-Synuclein Purification and Ionic Strength Modification Pivotal to Seed Amplification Assay Performance and Reproducibility

**DOI:** 10.3390/ijms25115988

**Published:** 2024-05-30

**Authors:** Chelva Janarthanam, Griffin Clabaugh, Zerui Wang, Bradley R. Melvin, Ileia Scheibe, Huajun Jin, Vellareddy Anantharam, Ramona J. B. Urbauer, Jeffrey L. Urbauer, Jiyan Ma, Arthi Kanthasamy, Xuemei Huang, Vincenzo Donadio, Wenquan Zou, Anumantha G. Kanthasamy

**Affiliations:** 1 Center for Neurological Disease Research, Department of Physiology and Pharmacology, University of Georgia, Athens, GA 30602, USA; chelva.janarthanam@uga.edu (C.J.); griffin.clabaugh@uga.edu (G.C.); ileia.scheibe@uga.edu (I.S.); huajun.jin@uga.edu (H.J.); vellareddy.anantharam@uga.edu (V.A.); ak39563@uga.edu (A.K.); 2 Department of Pathology, Case Western Reserve University School of Medicine, Cleveland, OH 44106, USA; zxw488@case.edu; 3 Department of Biomedical Sciences, Iowa State University, Ames, IA 50011, USA; bradmelvin00@yahoo.com; 4 Department of Chemistry, University of Georgia, Athens, GA 30602, USA; ramonau@uga.edu (R.J.B.U.); urbauer@uga.edu (J.L.U.); 5 Chinese Institute for Brain Research, Beijing 102206, China; majiyan@cibr.ac.cn; 6 Department of Neurology, Pennsylvania State University College of Medicine, Hershey, PA 17033, USA; xuh15@psu.edu; 7 IRCCS Institute of Neurological Sciences of Bologna, Complex Operational Unit Clinica Neurologica, 40138 Bologna, Italy; vincenzo.donadio@unibo.it

**Keywords:** Parkinson’s disease, pathological α-synuclein, αSyn, seed amplification assay (SAA)

## Abstract

Alpha-synuclein seed amplification assays (αSyn-SAAs) have emerged as promising diagnostic tools for Parkinson’s disease (PD) by detecting misfolded αSyn and amplifying the signal through cyclic shaking and resting in vitro. Recently, our group and others have shown that multiple biospecimens, including CSF, skin, and submandibular glands (SMGs), can be used to seed the aggregation reaction and robustly distinguish between patients with PD and non-disease controls. The ultrasensitivity of the assay affords the ability to detect minute quantities of αSyn in peripheral tissues, but it also produces various technical challenges of variability. To address the problem of variability, we present a high-yield αSyn protein purification protocol for the efficient production of monomers with a low propensity for self-aggregation. We expressed wild-type αSyn in BL21 *Escherichia coli*, lysed the cells using osmotic shock, and isolated αSyn using acid precipitation and fast protein liquid chromatography (FPLC). Following purification, we optimized the ionic strength of the reaction buffer to distinguish the fluorescence maximum (Fmax) separation between disease and healthy control tissues for enhanced assay performance. Our protein purification protocol yielded high quantities of αSyn (average: 68.7 mg/mL per 1 L of culture) and showed highly precise and robust αSyn-SAA results using brain, skin, and SMGs with inter-lab validation.

## 1. Introduction

Alpha-synuclein seed amplification assays (αSyn-SAAs) are a class of ultrasensitive diagnostic assays with the capacity to effectively distinguish Parkinson’s disease (PD) patients from healthy controls and to differentiate PD from other related synucleinopathies. PD emerges as a consequence of dopaminergic neuronal degeneration in the substantia nigra and is characterized postmortem by the presence of αSyn-rich Lewy bodies and Lewy neurites within the substantia nigra [1]. Recent studies indicate that αSyn seeding activity in the cerebrospinal fluid (CSF) can be used as a reliable biomarker for the diagnosis of synucleinopathies [2,3]. Evidence also suggests that αSyn pathology extends into the peripheral nervous system and can be detected in biomatrices such as the submandibular gland (SMG), skin, gastrointestinal tract, and serum [4,5,6,7,8,9,10,11,12]. Interestingly, αSyn pathology has also been detected in the periphery prior to the clinical motor manifestation of the disease [8,13], offering the possibility of minimally invasive early detection methods using SAAs seeded with peripheral biospecimens. Our group and others have shown that skin and SMG hold considerable promise for being used in SAAs with high diagnostic sensitivity and specificity [14,15,16,17,18,19,20].

SAAs utilize the propensity of misfolded αSyn to seed protein aggregation into amyloid fibrils in the presence of excess monomeric αSyn. This process occurs in a nucleation-dependent, self-propagating manner [21,22]. The SAAs involve regular cycles of shaking followed by quiescent (resting) incubation, which triggers the polymerization of pathological αSyn in vitro using only a minimal amount of sample to seed the reaction. Mechanistically, biospecimens containing misfolded αSyn seeds likely recruit and convert the monomeric substrate into beta sheet-rich amyloid fibrils during the resting phase. The subsequent shaking period mechanically breaks the aggregates into seeding-competent fragment templates that recruit and convert more αSyn substrate during the next resting incubation. The cyclic fragmentation and resting phases exponentially amplify the number of αSyn aggregates in the solution, which can be detected by Thioflavin T (ThT) fluorescence [22,23,24]. Thus, if the initial patient biomatrix contains misfolded αSyn, aggregation will occur rapidly (often within 24 h), while the absence of pathological αSyn will significantly delay aggregation.

The ultrasensitive nature of SAAs affords the capability of detection down to femtogram concentrations of misfolded, pathological αSyn in peripheral tissues. However, this degree of sensitivity also creates numerous technical challenges. Subtle changes in the recombinant αSyn purification protocols, αSyn monomer mutations, reaction mixture composition, pH and ionic strength of the buffer, tissue processing, shaking frequency, temperature, and plate reader settings appear to contribute to the inter-plate, inter-batch, and inter-lab variability of SAA results. These variables are often cited in inter-lab studies as potential reasons for discordance among results and remain a central problem in the SAA field [3,19,25]. Although this is inherent to many biological methodologies, the clinical implementation of SAAs will require analytical validation and standardization of critical components of the protocol. To this end, we adopted the nomenclature and recommended protocols outlined in the International Council for Harmonization (ICH) guidelines for the Validation of Analytical Procedures Q2 (R2) to assess the repeatability, intermediate precision, reproducibility, and robustness of the SAA aggregation kinetics. To make steps towards standardization, it is imperative that the protein purification methods and reaction buffer conditions yield SAA aggregation kinetics that are both precise and robust.

Monomeric αSyn is a major component of the SAA reaction mixture, but researchers use numerous sources of αSyn including proprietary options and various in-house purification methods. Proprietary αSyn monomer options have been used in SAA publications to support assay reproducibility [17,19,26], but could prove prohibitively expensive in a high-throughput setting. Therefore, in-house purification methods are needed to efficiently produce αSyn at a low cost with maximized yield, purity, and reproducibility for downstream SAAs. Generally, protein purification protocols use *Escherichia coli* (*E. coli*) expression systems to produce recombinant human αSyn, followed by cell lysis and fast protein liquid chromatography (FPLC). The details of cell lysis and FPLC vary depending on the lab and purpose of the αSyn product. In the context of SAAs, the protocol necessitates steps that reduce the likelihood of spontaneous αSyn seed formation and enhance monomer purity. However, batch-to-batch variations and low protein yield have been major challenges in the αSyn assay development.

In this study, we addressed the problem of SAA variability by introducing a detailed account of a high-yield αSyn purification method that yields an average of 68.7 mg/mL per 1 L of culture to support laboratories using single batches of protein for large SAA studies. Each protein batch was biophysically characterized prior to SAA validation by using a variety of methods to ensure the purity and uniformity of the monomer at the molecular scale. We also suggest alterations to the ionic strength of the reaction mixture for optimization, accompanied by using known controls and statistical measures to assess repeatability. Schematic 1 shows a detailed graphic of each step presented here. Together, we present these strategies to enhance the inter-batch and inter-lab repeatability/reproducibility of SAA kinetic metrics while maintaining the robustness of the assay when applied to biomatrices sourced from the brain, SMG, and skin.

## 2. Results

### 2.1. Our Optimized αSyn Protein Purification Demonstrates Inter-Batch Repeatability of Purity, Stability, and High Yields

**Figure 1:**
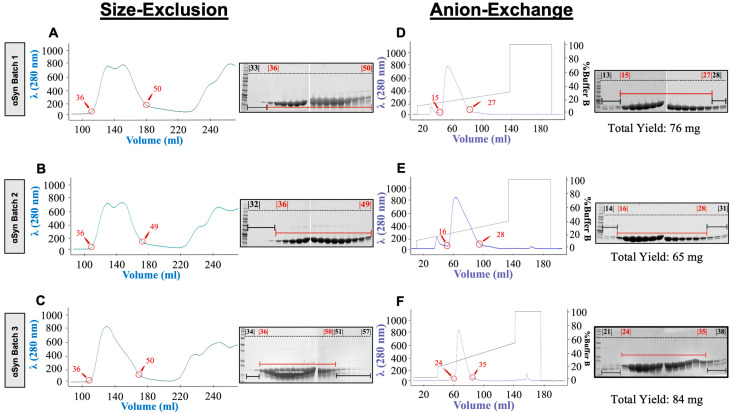
FPLC chromatographs from three representative αSyn batches show the highly repeatable purification of αSyn protein. (**A**–**C**) Size-exclusion chromatography (SEC) performed with absorption monitoring at 280 nm for all 3 αSyn monomer batches and the corresponding SDS-PAGE with Coomassie blue staining of SEC fractions taken for αSyn Batch 1, Batch 2, and Batch 3. The collected fraction ranges are indicated by red circles in the chromatographs, corresponding to the red underlined/overlined gel lanes. The black gel lanes denote the fractions that were discarded. (**D**–**F**) Fractions with an apparent molecular weight of 15 kDa contained αSyn and were pooled from the SEC fractions and further purified using anion-exchange chromatography and corresponding SDS-PAGE analysis of the collected anion-exchange fractions stained with Coomassie blue. The total yield of αSyn for each batch is given under the associated gel.

The purification of the αSyn monomer plays a vital role in the efficacy of SAAs with lysis protocols, with even small nuances of sample handling contributing to potential variability. Therefore, we sought to ensure a consistent level of repeatability with each batch of purified αSyn through various biophysical characterization methods. WT monomeric αSyn was purified with the bacterial expression protocol described in the Materials and Methods section. Following bacterial cell lysis via osmotic shock, acid precipitation, concentration, and filtering, the supernatant from each batch was injected into the FPLC instrument for gel filtration (Figure 1A–C). Purification consistency was qualitatively evaluated by comparing FPLC chromatographs and the resulting gel electrophoresis fractions. Fractions containing αSyn were determined by SDS-PAGE with Coomassie staining of the fractions under the largest absorbance peak. To maintain consistency across batches, only lanes with substantial amounts of αSyn were carried into the next step, as determined by SDS-PAGE. The selected lanes are denoted by red circles in the chromatographs and red underlined/overlined lanes in the SDS-PAGE. The remaining fractions were discarded. Collected fractions were then injected into the anion-exchange column of the FPLC instrument for each of the three batches (Figure 1D–F). Fractions with αSyn were established by SDS-PAGE with Coomassie staining, focusing on the band at approximately 14 kDa. The lanes with the most αSyn protein present were pooled while the rest were discarded. With reproducibility at the forefront of this work, we have highlighted the exact fractions taken from each batch and visualized the slight differences between the chromatographs. While minor variations arose between peaks and gel images, none of these deviations appeared to be an outlier. Following FPLC, the total yield of the purified protein was measured using a NanoDrop A280 with an extinction coefficient set at 5960 M^–1^ cm^–1^. The average total yield was 68.65 ± 12.26 mg, with the highest and lowest yields being 84 mg and 45 mg, respectively (Table 1).

**Figure 2:**
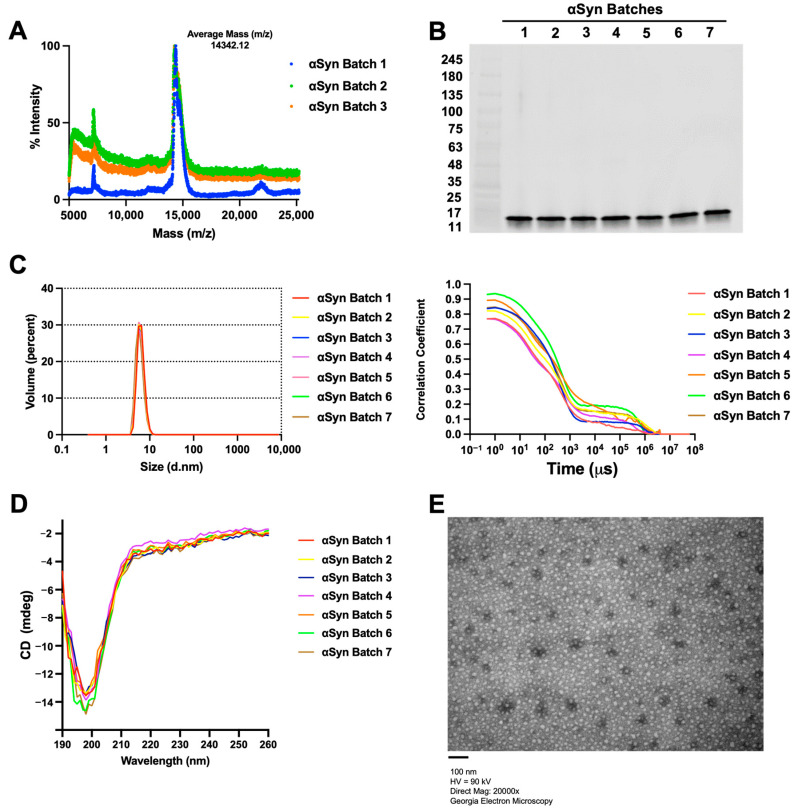
The biophysical characterization of the monomer gives evidence of its high purity and stability. (**A**) MALDI-TOF mass spectrometry results for 3 batches of αSyn monomers. (**B**) Western blot analysis of 30 µg of our in-house monomer, probing for total αSyn. (**C**) Dynamic light scattering (DLS) data illustrating size distribution by volume (left panel) and autocorrelation functions for different αSyn monomer batches (right panel). (**D**) Circular dichroism spectra of each batch of αSyn monomer. (**E**) Representative transmission electron microscopy (TEM) image of the αSyn monomer (scale bar = 100 nm).

Following the purification process, the αSyn monomer was characterized using MALDI-TOF mass spectrometry to give evidence for the presence of monomeric αSyn at the molecular weight of 14.4 kDa. The average molecular mass of all three batch monomers was 14,342.12 *m*/*z* (14.3 kDa), which confirms the molecular weight of monomeric αSyn with batch-to-batch repeatability in its relative mass intensity (Figure 2A). Additionally, an SDS-PAGE immunoblot using an antibody for total αSyn was conducted on seven batches of monomers, resulting in a singular band that further validated the αSyn identity of the purified monomer (Figure 2B). As a fundamental requirement for downstream SAA analysis, the purity of the αSyn monomers was evaluated by DLS. Size distribution by volume showed consistent particle sizes within all seven batches (Figure 2C, left panel) and the absence of other-sized molecules. A correlogram was generated using the Malvern Zetasizer software v8.00 (Malvern Panalytical, Great Malvern, England) showing the absence of randomness among the samples and supporting the volume-based size distribution data of the DLS (Figure 2C, right panel).

We next employed CD spectra to determine the secondary structure of each αSyn sample (Figure 2D). A strong negative band was visible near or below 200 nm (Figure 2D), which is indicative of a disordered protein [27]. Notably, the CD spectra of all monomer samples were essentially identical, confirming that all the protein monomers were disordered as expected for monomeric αSyn. This was further confirmed by estimating the percentage of secondary structural elements within these proteins based on their CD spectra using the CDSTTR algorithm available from the DichroWeb server. These results indicate that each batch monomer existed as predominantly disordered, with relatively insignificant amounts of ordered (helix, strand/sheet) secondary structure (Table S3).

Having confirmed the intrinsically disordered nature of our purified αSyn monomers by CD spectra, we further conducted TEM to visualize the molecular morphology of the αSyn protein in our various batches and to confirm the absence of any pre-formed fibrils (PFF)s during prolonged storage. The representative image (Figure 2E) was from a batch that had been stored for six months at −80 °C in 1× PBS. TEM images showed the absence of any fibril formation among the spherical monomeric population, confirming the stability of the monomer for subsequent SAA analysis. To validate this, an aliquot of the monomer was used to make PFF following the MJFF protocol, and the resulting PFF showed positive amplification in the SAA while the monomer alone did not (Figure S3). Together, these results suggest our protein purification method is highly repeatable and produces αSyn with consistently high purity, stability, and protein yield.

### 2.2. Ionic Strength Optimization Enhances the Fidelity of Fmax between Positive and Negative Controls

**Figure 3:**
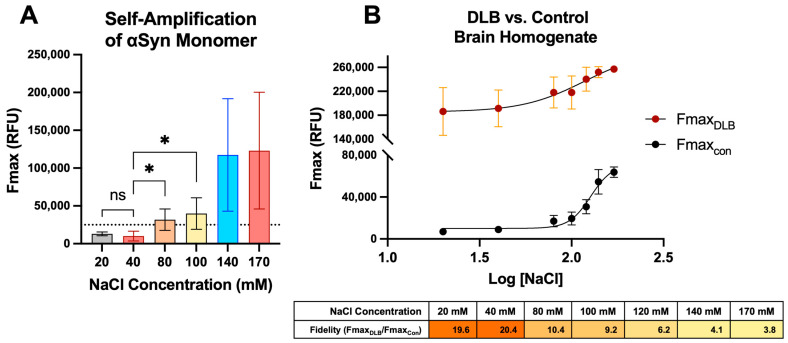
Ionic strength optimization of SAA reaction mixture reduces self-amplification and enhances fidelity. (**A**) αSyn monomer self-amplification in varying concentrations of NaCl after 40 h as measured by Fmax. The dotted line indicates the fluorescence threshold cutoff. (**B**) Mean Fmax RFU seen in the healthy control brain homogenate (*n* = 1) and DLB-positive brain homogenate (*n* = 1) as a function of NaCl concentration (Top Panel). Calculation of Fmax fidelity between positive and negative controls (bottom panel). * *p* ≤ 0.05; ns, not significant.

Differentiating between diseased and healthy control tissue matrices based on ThT aggregation curves is the principal goal of diagnostic SAAs. The effectiveness of this discrimination may be largely dependent on the reaction mixture conditions used to catalyze the aggregation process. In the field of synucleinopathy SAAs, all reaction mixtures contain monomeric αSyn in a buffer formulated for pH stabilization. From this point, many labs diverge by using different salts, detergents, buffers, and a range of concentrations of each constituent [17,21,25,28,29]. In our experience with SAAs, we have found the ionic strength of the reaction mixture to be the most influential factor for increasing assay fidelity, which is consistent with other reports [30,31,32]. For this reason, following monomer purification and biophysical characterization, the NaCl concentration of the reaction mixture was increased from 20 to 170 mM while keeping all other components constant to establish a reaction mixture with minimal self-amplification. Preliminary analysis showed a non-significant change in self-aggregation at NaCl concentrations greater than 170 mM (Figure S1). Using several batches of the αSyn monomer, we consistently found that the reaction mixture with 20–40 mM NaCl did not amplify above the threshold line over a 40 h period (Figure 3A). In contrast, our reaction mixture with ≥80 mM NaCl exhibited monomer self-amplification and crossed the threshold within 40 h. Statistically significant differences between the 20 or 40 mM and the 80 or 100 mM Fmax were observed across multiple batches of αSyn purified monomer (Figure 3A). Next, we sought to maximize the fidelity between DLB-positive and -negative control brain homogenates. The NaCl concentration was again increased from 20 mM to 170 mM while all other factors were held constant. The mean ThT Fmax, measured in relative fluorescence units (RFU), was recorded for the negative healthy control (Fmax_con_) and the DLB-positive control (Fmax_DLB_) at each concentration. The highest fidelity was achieved at 40 mM NaCl (Figure 3B), which was, therefore, selected as the optimized ionic strength for the reaction mixture in future assays.

### 2.3. The Combination of αSyn Purification and Ionic Strength Optimization Results in High Intermediate Precision and Repeatability in SAAs

The widespread implementation of the SAA requires the assay to have a high-level precision, which is defined here as low variation among technical replicates (repeatability) and across different αSyn batches (intermediate precision). Ideally, all technical replicates from a given sample should have similar aggregation kinetics (e.g., four out of four replicates amplifying simultaneously); however, this is not often the case in practice. Conflicting amplification from technical replicates makes it difficult to determine if the sample should be predicted as positive or negative. Replicate variability might stem from the assay’s ultrasensitive nature, posing an ongoing problem in the field. Furthermore, the SAA results should be repeatable, meaning the aggregation kinetics from a sample should show a high degree of inter-batch and inter-assay consistency. Thus, under our optimized conditions, we sought to evaluate the intermediate precision and repeatability across multiple batches of αSyn protein using various tissue matrices.

**Figure 4:**
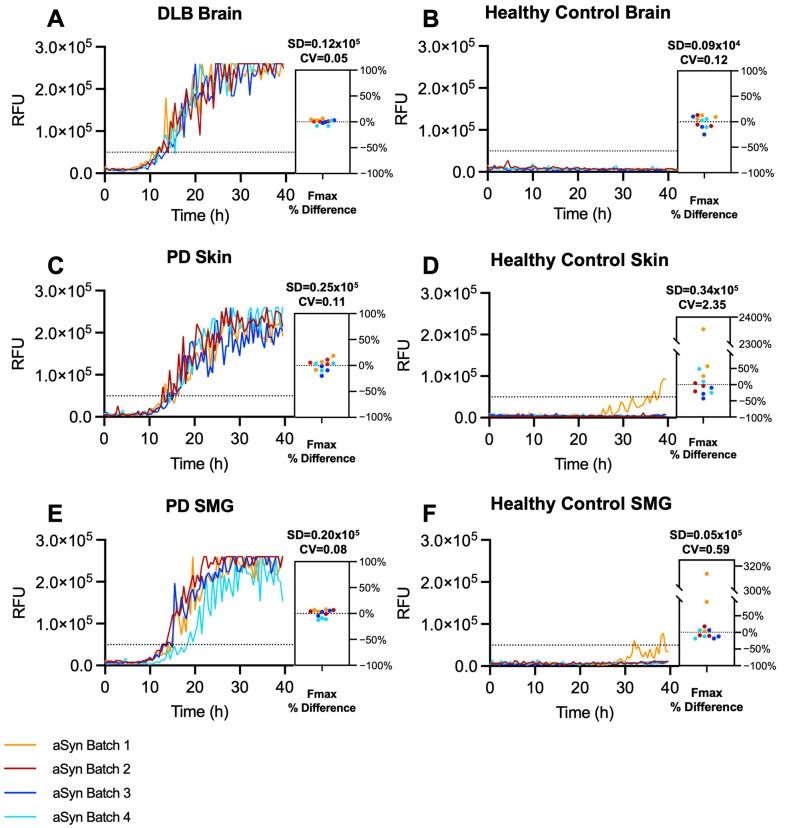
SAA from four αSyn batches using salt modification are repeatable and precise across tissue types. (**A**) ThT fluorescence over time from the brain homogenate of an individual with autopsy-confirmed DLB. (**B**) Brain homogenates from a healthy control. (**C**) Skin autopsy homogenates from an individual with autopsy-confirmed PD. (**D**) Skin homogenates from a healthy control. (**E**) SMG homogenates from an individual with PD. (**F**) SMG homogenates from a healthy control. The dotted line in each SAA curve indicates the fluorescence threshold cutoff. The scatter box plots display the individual replicates of each sample using different batches of αSyn monomer.

We employed four different batches of our monomeric αSyn protein produced on four separate days and by two different analysts. Three distinct tissue types (brain, skin, and SMG) were used to seed the reactions, with each tissue type having one known positive homogenate and one known negative homogenate used for seeding the reactions in triplicate. All tissue-positive samples were strongly amplified, and most control replicates did not amplify (Figure 4). To measure precision and repeatability, we first used a five-parameter fit function to account for noise and to acquire consistent measures for downstream analysis. Once the curve was fitted, the Fmax and PAR were derived from Equation (1). Variability was expressed using SD and coefficient of variation (CV) to evaluate the absolute and relative variability between replicates and across batches. The SD and CV were used for the positive and negative controls, respectively. Each aggregation curve is adjacent to a dot plot showing the percentage difference in Fmax of each replicate from the median to visualize replicates contributing to the observed variability. Kinetic parameters remained notably consistent across batches of the same sample as demonstrated by low CV values in the positive samples and comparable SD values in the negative controls. One exception was observed in the amplification of one replicate in the healthy control SMG sample and one replicate in the healthy control skin sample using αSyn Batch 1 (Figure 4D,F). As previously mentioned, the spontaneous aggregation of a single replicate is common in SAAs, and such instances are classified as negative based on our lab’s diagnostic criteria because the two other replicates did not cross the threshold. There were statistically significant differences between the positive and negative controls in all three tissue types (Table S4) with low variability of SAA kinetic parameters (Figure 4 and Figure S4). Additionally, we utilized skin biopsies obtained from PD and control subjects to determine the long-term stability of a batch of purified αSyn. We found similar aggregation kinetics of the PD sample over the course of six months of storage at −80 °C (Figure S5). These results show high intermediate precision and repeatability when using brain, skin, and SMG with the optimized reaction buffer conditions.

### 2.4. Comparative Assessment of SAA In-House WT Monomer and Proprietary K23Q Mutant Using Skin Autopsy Samples

**Figure 5:**
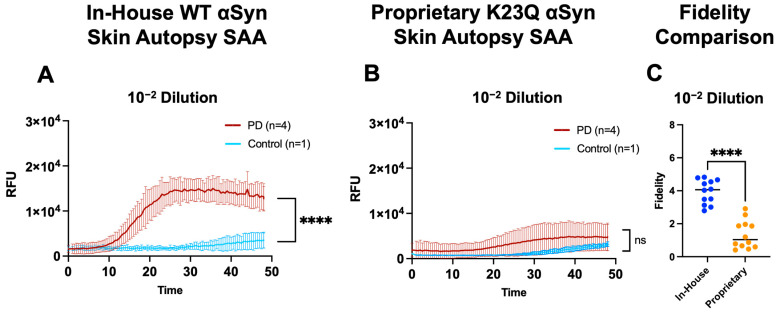

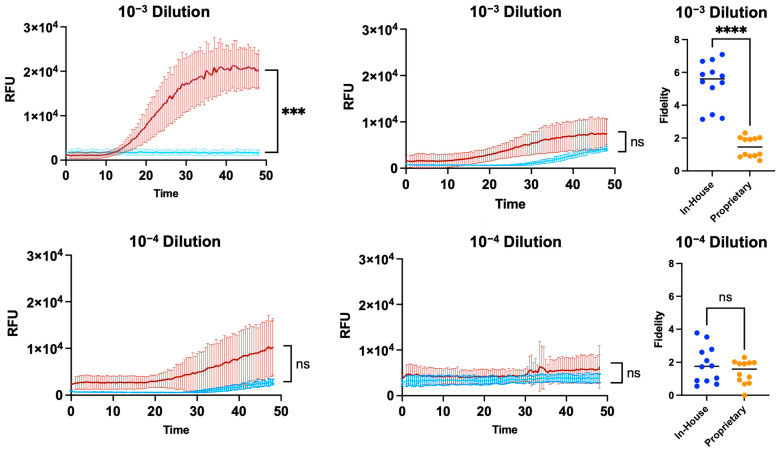
Comparison of SAA with skin autopsy samples using in-house WT αSyn vs. proprietary K23Q αSyn. (**A**) Seeding activity (48 h) of autopsy skin samples from neuropathologically confirmed PD (*n* = 4) vs. control (*n* = 1) samples diluted at 10^−2^, 10^−3^, and 10^−4^ using the in-house WT αSyn. (**B**) Seeding activity of biopsy skin samples from the same neuropathologically confirmed PD (*n* = 4) samples diluted at 10^−2^, 10^−3^, and 10^−4^ using the proprietary K23Q mutant monomer (Impact Biologicals Inc., Swarthmore, PA, USA). (**C**) Comparison of the fidelity of the in-house WT vs. the fidelity of the proprietary K23Q mutant. *** *p* < 0.001, **** *p* < 0.0001; ns, not significant.

Several groups utilize monomeric αSyn from proprietary sources for their SAA applications [17,19,26]. Although this strategy has proved effective, in our hands, we encountered challenges when applying these proprietary options to tissue matrices such as skin. Moreover, proprietary αSyn may be prohibitively expensive for many labs to implement in a high-throughput setting. To obtain a practical SAA comparison between our in-house WT αSyn monomer and a proprietary monomer, we used post-mortem PD (*n* = 4) and control (*n* = 1) skin autopsy samples in dilutions of 10^−2^, 10^−3^, and 10^−4^ tested in quadruplicates using a reaction mixture containing either our in-house WT monomer or the proprietary K23Q monomer. From the 48-h assay, the in-house WT monomer showed significant differences in the Fmax between the PD and control samples for the 10^−2^ and 10^−3^ dilutions (Figure 5A). In contrast, there were no significant differences in Fmax using the K23Q monomer in any dilution (Figure 5B). These results show that our WT monomer was highly sensitive with significantly higher fidelity in the 10^−2^ and 10^−3^ dilutions compared to the proprietary K23Q monomer, both tested on the same plate with skin autopsy samples (Figure 5C). The WT monomer yielded higher Fmax values, fidelity, and PAR among all positive samples compared to the K23Q mutant while maintaining the specificity of the assay. This result was repeated with another proprietary WT monomer under our SAA reaction conditions with brain samples (Figure S6). Collectively, our results show strong amplification of PD-positive skin autopsy samples with limited amplification of negative controls when compared to proprietary options.

### 2.5. External Lab SAA Validation Demonstrates High Reproducibility and Robustness of Our In-House Monomer

**Figure 6:**
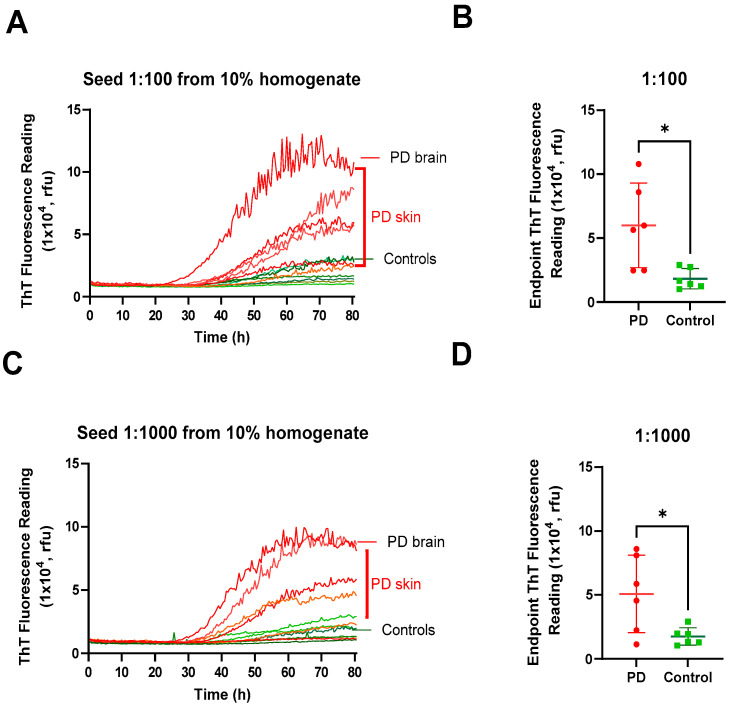
External lab validation of αSyn SAA demonstrates strong reproducibility. (**A**) The SAA kinetic spectrum of the ThT fluorescence of autopsy brain and skin samples from neuropathologically confirmed PD (*n* = 6, red) and non-PD (*n* = 6, green) diluted at 1:100. (**B**) Scatter plot and statistical evaluation of the endpoint ThT fluorescence of the SAA assay shown in panel A. (**C**) SAA kinetic spectrum of ThT fluorescence of autopsy brain and skin samples from neuropathologically confirmed PD (*n* = 6, red) and non-PD (*n* = 6, green) diluted at 1:1000. (**D**) Scatter plot and statistical evaluation of the endpoint ThT fluorescence of the SAA assay shown in panel C. * *p* ≤ 0.05.

Next, we assessed the robustness and reproducibility of our in-house WT monomer by providing it to a second laboratory (the Zou Laboratory) for validation. The reaction mixture conditions used in the Zou laboratory were distinct from our own, offering a valuable opportunity to evaluate the robustness of the assay. Their alterations to the assay conditions included variations in plate reader shaking intervals and the addition of an N2 supplement. The positive αSyn seeding activity (SA) was observed in both PD skin and brain samples when diluted at ratios of 1:100 (Figure 6A,B) and 1:1000 from 10% homogenate (Figure 6C,D). In contrast, no positive SA was observed in the non-PD control samples. In addition, the Fmax reading (Figure 6B,D) enabled the differentiation of PD from the non-PD controls (*p* < 0.05). The SA kinetic curves exhibited a shorter lag phase and a higher Fmax intensity in the brain than in the skin (Figure 6A,C), which was more obvious at a lower dilution of the samples. Despite significant variations in the plate reader shaking setting and minor differences in the sample buffer, our in-house monomer was capable of distinguishing between positive and negative brain and skin homogenates. Overall, these results establish our in-house monomer’s capacity to provide reproducible and robust SAA aggregation kinetics.

### 2.6. Blinded Skin Biopsy SAA Validates High Sensitivity and Specificity

**Figure 7:**
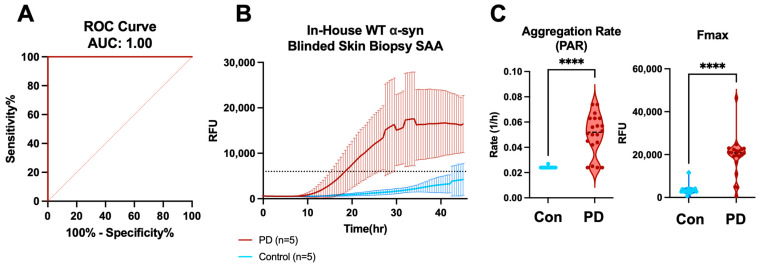
In-house αSyn differentiates between PD and control skin biopsies in our optimized SAA conditions. (**A**) ROC curves for skin biopsy SAA comparing the PD and control groups using our algorithm scoring system. (**B**) Seeding activity of biopsy skin samples from clinically diagnosed PD (*n* = 5) vs. control (*n* = 5) samples diluted at 10^−2^ using in-house WT αSyn. The dotted line represents the fluorescence threshold cutoff. (**C**) The PAR of each replicate in the PD vs. control skin biopsy samples (left, each dot represents a replicate in the violin plot) and Fmax of each replicate in the PD vs. control skin biopsy samples (right, each dot represents a replicate in the violin plot). **** *p* < 0.0001.

Recent studies from our group and others have confirmed the ability to use peripheral tissue matrices to seed the aggregation reaction in SAAs with high sensitivity and specificity [12,14,15,16,17,18,19]. In our recent publications, we demonstrated skin as a promising, readily accessible tissue that can be obtained by a minimally invasive procedure for seeding the reaction with an impressively high sensitivity and specificity [14,33]. To determine the performance of our in-house monomer and optimized conditions using antemortem skin biopsies, we executed a blinded study involving a small cohort of PD (*n* = 5) and control (*n* = 5) skin biopsies. Using the optimized 10^−2^ dilution, we tested all 10 samples in quadruplicate for αSyn seeding activity and utilized the algorithm described in the Supplementary Materials to establish the positivity of the blinded samples. Following the SAA procedure and data analysis, the results were sent for unblinding. After unblinding the SAA results, we correctly identified all five PD and five control samples (100% sensitivity, 100% specificity) as measured by the receiver operating characteristic (ROC) (Figure 7A). Nearly all PD replicates started amplifying before 20 h and exhibited statistically higher PARs and Fmax intensities than control samples (Figure 7B,C). These results, consistent with our previous observations [14,33], suggest our WT in-house monomer, combined with optimized ionic strength conditions, is highly suitable for peripheral biomatrices such as skin, in terms of being able to significantly differentiate between PD and healthy control patients.

## 3. Discussion

The incorporation of seeding aggregation methods into the diagnostic pipeline of PD and related synucleinopathies continues to show promise. To make steps towards clinical implementation, there is a need to ensure the assay is highly precise, repeatable, reproducible, and robust. Inevitable challenges emerge when different labs use a multiplicity of tissue processing methods, protein purification protocols, and reaction mixtures. Here, we have shown a high-yield protein purification protocol, quality control procedures, and ionic strength optimization to ameliorate the problem of SAA variability. Our objective was to isolate monomeric αSyn using gentle cell lysis techniques with the final product possessing minimal contaminants or template seeds to reduce the likelihood of spontaneous aggregation in downstream assays. To this end, we used osmotic shock lysis, acid precipitation, and FPLC for purification, incorporated streptomycin to eliminate nucleic acid contamination, and conducted extensive dialysis to ensure low ionic strength during storage and subsequent SAAs.

Recombinant αSyn protein purification protocols use an expression vector and specialized bacterial cell lines, such as BL21 (DE3) cells, to express the protein in *E. coli* followed by cell lysis. Many strategies have been devised to lyse bacterial cell membranes and are broadly categorized as mechanical and non-mechanical methods as reviewed in [34,35]. Non-mechanical, physical, and chemical lysis processes are frequently used for αSyn purification, including osmotic shock, sonication, and chemically induced lysis. Osmotic shock works by incubating the cells with a hypertonic solution to weaken the outer membrane and subsequently adding a hypotonic solution to burst the outer membrane to extract the periplasmic proteins while maintaining the integrity of the inner membrane [35]. This may decrease the likelihood of cytoplasmic protein contaminants and minimize αSyn misfolding arising from sonication. Following cell lysis, additional steps like boiling, acid precipitation, or (NH_4_)_2_SO_4_ precipitation can be used to further isolate αSyn before FPLC. Although beneficial to purification, these precipitation steps can alter intermolecular bonding and influence the conformational dynamics of the monomer. In the context of SAAs, acid precipitation appears to yield αSyn that is less prone to self-aggregation compared to other methods [36], which is further supported in this work.

Osmotic shock is an effective technique for selectively extracting periplasmic proteins such as αSyn, while maintaining the integrity of the inner membrane to avoid potential contaminants [37,38]. Acid precipitation is a process of transiently decreasing the pH of the solution to precipitate undesired proteins and retaining the soluble fraction containing αSyn. Alternatively, sonication and boiling are common methods used for αSyn protein extraction, but these methods may promote αSyn seed formation by increasing the entropy of the system [39,40]. Based on previously published work [36,37,41] and the comparatively gentle nature of the methods, osmotic shock and acid precipitation working in tandem were hypothesized to generate monomeric αSyn with high purity and stability for reproducible downstream SAA aggregation kinetics. We also introduced streptomycin to the supernatant after precipitation to eliminate potential nucleic acid contaminants, which could otherwise contribute to variability. Moreover, two 12 h H_2_O dialysis incubations were used to eliminate NaCl from the microenvironment, because the presence of even low concentrations of ions can contribute to heterogenous αSyn morphologies and may induce the formation of αSyn seeds [42]. With ionic strength being a crucial component of reaction mixture optimization, maintaining it at a constant level was of paramount importance and may contribute to repeatable results. Overall, using osmotic shock and acid precipitation, our modified protein purification protocol reported here consistently yielded large quantities of highly pure αSyn and provided strong inter-batch repeatability in downstream SAA analyses.

In conjunction with monomer purification and αSyn mutations, the physicochemical properties of the solution conditions have a major impact on αSyn aggregate morphology and aggregation kinetics [31,32,43,44]. Altering the reaction mixture pH, temperature, ionic strength, detergents, and metal ions can contribute to variations in αSyn fibrilization [40,45]. The ionic strength and ion type have a pronounced impact on fibrillation kinetics and fibril strain due to long-range electrostatic interactions of the negatively charged C-terminal and positively charged N-terminal of αSyn [42,44]. Roeters et al. [42] proposed a model based on 2D-IR spectroscopy experiments whereby monomeric αSyn exhibits a compact structure under NaCl concentrations less than 25 mM, with the N- and C-terminals shielding the aggregation-prone NAC region, resulting in a more extended, ribbon-like fibril conformation. In contrast, starting between 25 and 50 mM of NaCl, a conformational shift was observed due to Na^+^ and Cl^−^ ions interacting with the C-terminal and N-terminals, respectively. This electrostatic shielding may expose the hydrophobic NAC region, inducing transient intramolecular β-sheet structures, leading to a higher likelihood of fibrilization with a twisting morphology [42].

In the context of SAAs, the optimal αSyn monomer microenvironment is one that produces a prolonged lag time in the absence of pathological αSyn, but quickly initiates aggregation upon exposure to seed templates. Our optimized 40 mM NaCl concentration is on the boundary between the reported intramolecular shift and may explain why our reaction mixture is primed for aggregation while preserving the extended lag time. Previous studies have demonstrated a similar optimization strategy by adjusting the salt concentration and selecting the appropriate Hofmeister species for specific diseases [26,30]. While 40 mM NaCl is less than that employed by many other SAA labs, factors including αSyn mutations, pH, detergents, and shaking settings may explain the differences. The ionic strength adjustments here reinforce the conclusion that the ionic strength of the reaction mixture is critical to the assay’s performance in distinguishing disease versus control samples.

These in vitro studies provide strong evidence that ionic concentration affects αSyn structure and aggregation kinetics. These findings may have mechanistic implications on how microenvironmental ions influence αSyn aggregation in vivo. αSyn is primarily localized to presynaptic terminals where it encounters ions, including Na^+^, K^+^, Ca^2+^, and Mg^2+^, all of which may affect its structure and aggregation rate. Under normal physiological conditions, the intracellular sodium concentration is 15 mM [46]. Interestingly, recent reports using sodium MRI demonstrate that patients with neurological conditions, including PD and Alzheimer’s disease, present with an increased intracellular sodium concentration and could be explored as a factor contributing to fibril formation in vivo [46,47,48].

Substantial diversity exists in the purified recombinant αSyn monomer used as substrate in SAAs. Proprietary αSyn proteins, such as those offered by Proteos, Impact Biologicals, rPeptide, and Stratech, have been used in several publications [17,21,26,49], while other labs achieve better results using in-house monomers under their specific conditions [14,28,29]. Proprietary monomer costs between 500–630 USD (rPeptide [Watkinsville, GA, USA] and K23Q Impact Biologicals Inc. [Swarthmore, PA, USA]) for 1 mg, and some assays in the SAA field use up to 3 mg for a single assay. This could pose a prohibitive economic burden when scaling up with larger cohorts. In comparison, an average of 68.65 ± 12.26 mg per batch from our protocol leads to significant cost savings when compared to procuring the least expensive proprietary monomer available in the field. Furthermore, numerous αSyn variants (i.e., WT, A53T, and K23Q) and distinct αSyn purification methods are used in the assays. Evidence shows specific mutants being more sensitive to self-amplification, while others may be more resistant to self-amplification. For example, the K23Q mutation generates comparable aggregation kinetics to WT but is less likely to spontaneously fibrillate [50], while A53T mutations may be more aggregation-prone [51]. Under our purification method and assay conditions, WT αSyn demonstrated stronger amplification in positive samples with minimal amplification in negative controls, as compared to K23Q mutant αSyn.

In addition to lab-to-lab differences in protocols, we acknowledge some potential limitations of the current SAA technology. The ultrasensitivity of the assay presents the possibility of false positives due to contamination. Thorough quality control steps and the implementation of strategies to reduce contamination are warranted to reduce the likelihood of false positives. Non-specific aggregation is also a commonly cited limitation of SAAs; however, the use of proper reagent controls and consistent αSyn purification protocols can ameliorate this problem. Moreover, the assay remains semi-quantitative without a way to accurately quantify the number of αSyn seeds in the sample. Synthetic PFFs cannot be employed to estimate the endogenous αSyn seeds in biomatrices, and efforts should be directed toward developing standards. 

Despite the limitations, considerable potential remains for SAAs to accurately diagnose PD. The clinical diagnosis of PD is currently based on motor and non-motor symptoms, along with levodopa response tests. Imaging methods, such as SPECT and PET, are used to support a differential diagnosis. However, the diagnostic accuracy of PD remains about 84% depending on the physician’s experience and length of follow-up [52]. Although diagnostic advancements have been made in the past decade, reliable diagnostic tools for PD are still lacking. Recently, the use of skin-, SMG-, and blood-based SAA methods have garnered significant traction due to their high diagnostic performance and minimally invasive collection methods [12,14,15,17,18,19,20,33]. Recent studies have demonstrated the robustness and reliability of SAAs for PD diagnosis. For example, a CSF-based SAA by Siderowf et al. [2] reported a diagnostic efficacy of 96.3% specificity and 87.7% sensitivity with a cohort of 1123 participants. Additionally, a serum-based SAA by Okuzumi et al. [12] demonstrated 91.5% specificity and 95% sensitivity. These two studies highlight the emerging robustness and reliability of SAAs for PD diagnosis even with diverse assay conditions and tissue types. With the reported accuracy in most of the SAA studies being greater than 90%, the prospect of implementing SAAs into the diagnostic criteria for PD could be on the horizon. However, considerable effort is still required to determine if SAAs can serve as early or prodromal biomarkers of PD, or if they can be used to track disease progression. Due to the limited sample size of this study, issuing firmer conclusions about the diagnostic accuracy of skin biopsies in SAAs should await results from our ongoing studies with larger populations. This small study can serve as a template to develop monomer purification pipelines with adequate quality control steps and strategies to optimize the ionic strength of the reaction mixture in many tissue types.

Systematic validation of the SAA analytical procedure is critical to making progress toward wide clinical implementation of the assay. Here, we used the defined terminology and suggested data parameters specified in the updated ICH Q2 (R2) Validation of Analytical Procedures Guidelines [53] to assess the precision and robustness of the assay with our in-house monomers. Once finalized, the ICH Guidelines will serve as the current thinking of the Food and Drug Administration (FDA) on approving analytical procedures for industry. Precision is broken down into three main categories: repeatability, intermediate precision, and reproducibility. Assessing the robustness of an analytical procedure involves deliberately introducing variation to the procedure. Our data demonstrated high levels of repeatability and intermediate precision as measured by low SD and CV in Fmax and PAR measurements across multiple monomer batches. We also showed the assay’s inter-lab reproducibility using our monomer to effectively distinguish between PD and healthy control subjects. Robustness is an inherent problem in ultrasensitive assays, with slight changes contributing to variability as discussed previously. In the inter-laboratory experiment, we deliberately introduced changes to the plate reader shaker settings and dilution mixtures to determine the robustness of the assay with our monomer. Although the alterations changed the rate of aggregation, we showed that our in-house monomer remained capable of differentiating between PD patients and control subjects with significantly different Fmax ThT fluorescence readings.

To remain consistent in data analysis, we also used the five-parameter fit model to accurately characterize several aspects of the aggregation kinetics. This approach allowed us to make semi-quantitative comparisons of each curve by using SD and CV. Using this model can also provide a high-throughput solution for calculating Fmax, PAR, fidelity, and hit rate. This capability proves useful not only for diagnostic applications of SAAs but also for the validation of new batches of protein and the calculation of inter-batch and inter-lab variability.

Due to the highly dynamic interplay between the αSyn purification method, reaction environment, and differences in sample handling, the implementation of SAAs into the synucleinopathy diagnostic pipeline has proved challenging. Overall, our findings underscore the crucial roles played by the purification protocol and ionic strength of the reaction mixture in ensuring the precision and robustness of the assay. While considerable work is still needed to improve the reproducibility of SAAs between laboratories, our purification method and ionic strength optimization may be valuable in the clinical standardization of SAAs using peripheral biospecimens.

## 4. Materials and Methods

### 4.1. Sample Collection

Autopsy Brain: We obtained deidentified frozen brain tissue collected from individuals diagnosed with dementia with Lewy bodies (DLB) and healthy control subjects obtained from UC Davis Alzheimer’s Disease Center. Upon arrival, these samples were stored at −80 °C. The biological samples were histologically analyzed using the standard criteria for diagnosis before testing in the SAA.

Autopsy Scalp and SMG: Samples of PD patients and healthy control individuals were purchased from Banner Health. The samples were stored at −80 °C upon arrival. Before being subjected to the SAAs, all these biological samples had been histologically analyzed using the standard criteria for diagnosis. 

Skin Biopsy: Samples from PD patients and healthy controls were obtained from the IRCCS Institute of Neurological Sciences of Bologna, Bologna, Italy. The samples were stored at −80 °C upon arrival. The biological samples were histologically analyzed using the standard criteria for diagnosis before testing in the SAA.

### 4.2. Autopsy Brain and SMG Homogenate Preparation

The tissues were weighed and cut into small pieces before adding a volume of 1× TBS (sterile) to make a 10% (*w*/*v*) solution in a 1.5-mL RINO tube. Next, 0.5-mm zirconium beads were added to the tube, and the mixture was homogenized using a bullet blender (BioSpec, New York, NY, USA) for 5 cycles of 1 min with a 1-min rest on ice between each cycle. The homogenate was centrifuged at 500× *g*, and the resulting supernatant was extracted and dispensed into 10-μL aliquots. These 10% homogenates were stored at −80 °C. We adopted the following nomenclature for serial dilutions: the initial 10% homogenate was considered 10^−1^ dilution; from the 10%, a 1:10 dilution resulted in 10^−2^ dilution; etc. 

### 4.3. Autopsy Skin and Biopsy Skin Homogenate Preparation

The tissues were weighed and a volume of tissue lysis buffer (2 mM CaCl_2_, 0.25% *w*/*v* Collagenase A [Millipore Sigma, Darmstadt, Germany]; Cat# 10103578001 in 1× TBS) was added to make a 10% (*w*/*v*) solution in a 1.5-mL Eppendorf tube. The sample was incubated in a thermomixer at 37 °C with 350-rpm shaking for 4 h. Next, the incubated samples were transferred into a 1.5-mL RINO tube along with 0.5-mm zirconium beads and homogenized using a bullet blender for 5 cycles of 1 min with a 1-min rest on ice between each cycle. The homogenate was centrifuged at 500× *g* and the supernatant was extracted and dispensed into 10-uL aliquots. The 10% homogenates were stored at −80 °C.

### 4.4. High-Yield Purification of Recombinant αSyn

The protein purification protocol was adopted from Groveman et al. [28] with minor modifications. Briefly, the αSyn protein was expressed in BL21 *E. coli* cells that had been transformed with a pT7-7 αSyn WT expression plasmid as previously described [54]. For this experiment, one aliquot of the bacterial cells, stored at −80 °C, was retrieved and thawed to prepare a 5-mL mini culture in lysogeny broth (LB, Millipore Sigma, Darmstadt, Germany; #L3022). The mini culture was grown at 37 °C with 230-rpm shaking for 8 h. Next, the mini culture was transferred to a pre-warmed 1-L LB culture supplemented with antibiotics, and the overnight autoinduction reagents (Millipore Sigma, Darmstadt, Germany; #71300-4) were then added as per the manufacturer’s instructions in a 2.5-L Tunair flask. Once the OD600 reached 1.2–1.3 (approximately 12 h), the 1-L culture was divided equally into 4 × 250-mL bottles (Cole-Parmer, Vernon Hills, IL, USA; #0603543). The bacterial cells were subsequently pelleted by centrifugation for 20 min at 3750 rpm at 4 °C, and the supernatant was discarded.

Room temperature (RT) osmotic shock buffer (100 mL—40% sucrose, 30 mM Tris HCl, 2 mM EDTA, pH 7.2) was prepared fresh and 25 mL was added to each of the 4 pellets obtained (pellet size was measured for quality control between batches) and resuspended gently using a 25-mL serological pipet, avoiding contact of the tip with the pellet. Once the suspension looked homogenous without visible pellet chunks, the suspension was transferred into four 50-mL tubes. The tubes were horizontally placed in a flat container and set on a rocker at a slow speed for 10 min at RT while avoiding bubble formation. Tubes were centrifuged at 9000× *g* at 20 °C for 20 min. The supernatant was discarded, and pellets were placed on ice. Each pellet was gently resuspended with a serological pipet in 25 mL of approximately 2 mM MgCl_2_ while avoiding contact of the tip with the pellet (MgCl_2_ was prepared as 100 mL ice-cold Milli-Q H_2_O + 40 µL 5 M MgCl_2_). Once the suspension was visibly homogenous with no pellet chunks, the tubes were gently placed on a flat container set on a rocker allowing it to roll back and forth for 5 min at 4 °C. The tubes were centrifuged at 9000× *g* at 4 °C for 30 min. The supernatant was collected in a 150-mL sterile glass beaker with a magnetic stir bar and kept immersed in ice. The beaker was placed on a stirrer and gently stirred as the pH was reduced to 3.5 using 1 M HCl (a white precipitate appeared, turning the solution white and cloudy) and continued stirring for 5 min. The solution contents were transferred into four 50-mL tubes and centrifuged at 9000× *g* at 4 °C for 30 min. The supernatant was collected in a 150-mL sterile glass beaker with a magnetic stir bar and kept immersed in ice. The beaker was placed on a stirrer and the solution was gently stirred as the pH was increased to 7.5 using 1 M NaOH. The contents were then transferred into two 50-mL tubes kept on ice. Streptomycin sulfate (Thermo Fisher Scientific, Waltham, MA, USA; 11860038) was added to make a final concentration of 2.5 mg/mL and gently placed on a flat container set on a rocker allowing it to roll back and forth for 10 min at 4 °C. The tubes were centrifuged for 20 min at 24,000× *g* at 4 °C. The supernatant was collected in two 50-mL tubes kept on ice. Before application to the FPLC column, the sample was concentrated from 100 mL to 15–20 mL using 3-kDa Amicon Ultra-15 Centrifugal Filters (Millipore Sigma, Darmstadt, Germany; UFC900324) at 4000× *g*. The sedimented precipitate at the bottom of the filter was resuspended and collected. Finally, the sample was filtered using a 0.22-µm syringe filter.

The size-exclusion chromatography (SEC) column, Sephacryl S-200 HR 26/60 (Cytiva, Marlborough, MA, USA; #17119501) was equilibrated with 3 to 5 column volumes (CVs) of SEC buffer (10 mM Tris HCl, 50 mM NaCl, 1 mM EDTA, pH 7.5) at a minimal flow rate of 1 mL/min setting overnight using the NGC Chromatography System (Bio-Rad Laboratories, Inc., Hercules, CA, USA). To achieve repeatable purification chromatographs, the columns were thoroughly cleaned once every two months. Next, 10 mL of the prepared sample was manually loaded into the 10-mL sample loop of the NGC chromatography system and systemically loaded into the column using 15 mL of the SEC buffer. The same was repeated for the remaining sample. The SEC protocol was initiated with the following protocol.

All buffers were prepared fresh, filtered using 0.4-µm rapid flow filters, and degassed. At a flow rate of 1 mL/min, 2 CVs of fresh SEC buffer were used to elute the target protein in 3-mL fractions followed by a non-fraction 3-CV equilibration stage to maintain the column for future use. The chromatogram showed a major absorbance peak associated with the target protein, and all fractions under the peak were collected and subjected to SDS-PAGE analysis. Coomassie stain was used to visualize the expression of αSyn at the expected 14.4-kDa molecular weight. In this protocol, we utilized a selection criterion for the fractions based on the SDS-PAGE. The fractions containing very little αSyn expression (as evidenced by the SDS-PAGE band at 14.4 kDa) were discarded, while fractions with strong αSyn expression were pooled and carried into anion-exchange chromatography.

First, 10 mL of the pooled fractions from size-exclusion chromatography was manually loaded into the 10-mL sample loop of the NGC chromatography system and subsequently loaded into the anion-exchange column, HiPrep Q FF 16/10 (Cytiva, Marlborough, MA, USA; #28936543) using 15 mL of low-salt Buffer A (25 mM NaCl, 10 mM Tris HCl, 1 mM EDTA, pH 7.5). The same was repeated for the remaining sample and the SEC protocol was initiated.

At a flow rate of 1 mL/min (the isocratic segment), 2 CVs of 5% high-salt Buffer B (1 M NaCl, 10 mM Tris HCl, 1 mM EDTA, pH 7.5) were pushed through the column without fraction collection. This was followed by a fraction collection elution stage using a flow rate of 1 mL/min with a 6-CV gradient from 20% to 55% Buffer B with collection of 2-mL fractions. An additional step consisting of 2 CVs of 100% Buffer B at a flow rate of 2 mL/min was pushed to elute non-target proteins bound to the column. A final equilibration stage using 3 CVs of Buffer A was added to maintain the column for future use. The chromatograph showed an absorbance peak associated with the target protein eluting at ~65 mL (~29% Buffer B). Fractions within the peak and two fractions immediately before and after the peak were collected for SDS-PAGE analysis. The Coomassie-stained gel confirmed the expression of αSyn. Like SEC, the fractions containing very little αSyn expression, as revealed by SDS-PAGE analysis, were discarded, while fractions with strong αSyn expression were pooled and carried into dialysis.

The selected fractions were pooled and loaded into snakeskin dialysis tubing (3.5 K MWCO, 16 mm, Thermo Fisher Scientific, Waltham, MA,USA; 88242) with clips. Air bubbles were avoided when sealing the tubing to prevent contamination. The protein was gently stirred using a magnetic stirrer and stir bar in pre-chilled 4 L MilliQ-H_2_O at 4 °C for 12 h. After 12 h, the water was replaced with 4 L of fresh MilliQ-H_2_O and dialyzed for an additional 12 h at 4 °C. The protein was then transferred into a 50-mL tube using a serological pipette. The concentration of the monomer was determined by measuring the absorbance at 280 nm using a NanoDrop spectrophotometer (Thermo Fisher Scientific, Waltham, MA, USA) employing a 340-nm baseline correction, assuming an extinction coefficient of 5960 M^−1^ cm^−1^ and a molecular weight of 14.4 kDa. The monomer was diluted to yield a final concentration of <1 mg/mL (approximately 0.7 mg/mL) using 40 mM sodium phosphate buffer pH 8. Aliquots were made in 1.5-mL low protein-binding tubes and stored at −80 °C.

### 4.5. SDS-PAGE

Once the fractions were collected, 10 µL of each fraction was mixed with an equal amount of 2× Laemmli sample buffer (Bio-Rad Cat #1610737) and heated at 98 °C for 10 min on a thermomixer. A 1× Tris/Glycine/SDS Buffer solution was prepared by mixing Milli-Q H_2_O and Bio-Rad 10× concentrate (Bio-Rad Cat # 1610772) to create a final concentration of 25 mM Tris, 192 mM glycine, and 0.1% (*w*/*v*) SDS, pH 8.3. Proprietary gels (Bio-Rad 10-well Any kD Mini-PROTEAN TGX) were loaded with a 10-µL protein ladder in the first well followed by 10-µL samples in the remaining 9 wells. With a Mini-PROTEAN Tetra Cell, 2-Gel System connected to a Bio-Rad Power Pac 1000, SDS-PAGE was run at a constant voltage of 120 V until the bands ran off the bottom of the gels. The unstained gels were carefully placed in a plastic container and washed with deionized (DI) H_2_O for 5 min, 3 times at RT on a shaker. Following washing, 25 mL of Coomassie blue stain (Bio-Rad Coomassie G-250 Cat# 1610786) was added to the gel and stained for 30 min with gentle rocking at RT. After staining, the gel was washed with DI H_2_O for 30 min and scanned using an LI-COR scanner.

### 4.6. Western Blot

For each batch of monomer, 30 µg of total protein was loaded onto an Any kD gel and run at 170 V at 4 °C. The protein was transferred to a nitrocellulose membrane with a setting of 0.22 amps for 1.75 h. The membrane was rinsed with water for a few seconds before blocking with a 50:50 blocking buffer (LI-COR blocking buffer PBS: 1× PBS) for 1 h. The membrane was probed with an αSyn primary antibody (BD Bioscience Purified Mouse Anti-α-synuclein AB_398107) at a 1:1000 concentration overnight at 4 °C. The membrane was washed 4 times for 5 min with PBST. A donkey anti-mouse secondary antibody was used to probe at 1:10,000 concentration for 1 h at RT followed by four 5-min washes with PBST and scanned using an LI-COR scanner.

### 4.7. Dynamic Light Scatter (DLS)

DLS analysis of the monomers was performed using the Zetasizer Nano-ZS (Malvern Panalytical, Great Malvern, England) instrument, housed in the Integrated Bioscience and Nanotechnology Cleanroom at the University of Georgia, and Zetasizer software version 8.2 was used to analyze the data. The monomers were spin-filtered using a 100-kDa filter. The measurement position was set to 4.65 mm and a wavelength of 633 nm. A sodium phosphate buffer was used as the dispersant with a refractive index setting of 1.332 and a viscosity of 0.8890. For the protein, the refractive index was set to 1.45, and absorption was set to 0.001.

### 4.8. MALDI-TOF Mass Spectrometry

To confirm the molecular masses of the monomers, we used matrix-assisted laser desorption ionization time-of-flight (MALDI-TOF) mass spectrometry for 3 different monomer batches. The monomers were spin-filtered using a 100-kDa filter and 1 µL of each sample mixed with 10 µL of the MALDI matrix 2,5-dihydroxybenzoic acid in triplicates were dispersed on a MALDI-TOF mass spectrometry target plate and analyzed.

### 4.9. Transmission Electron Microscopy (TEM)

TEM imaging was performed by placing a 400-mesh, formvar/carbo-coated copper grid over a 20-µL drop of the sample solution for 15 min (the monomer sample had been previously filtered using a 100-kDa spin filter). The excess sample solution was then wicked off with a filter paper wedge, and the grid was then placed on a 20-µL drop of 3% aqueous phosphotungstic acid (pH 7.0) for 30 s. After removing the excess stain, the sample was allowed to dry before imaging at 90 kV with a JEOL 1011 TEM (JEOL USA, Peabody, MA, USA). Images were taken with an AMT mid-mount camera (AMT, Woburn, MA, USA). Preformed fibrils (PFFs) were generated using the Michael J. Fox Foundation (MJFF) protocol for the generation of pre-formed fibrils from αSyn monomer.

### 4.10. CD Spectrum

Far-UV circular dichroism (CD) spectra of the protein samples were acquired using a Jasco J-715 spectropolarimeter (Jasco, Eason, MD, USA). The samples included 4 µM protein in 20 mM sodium phosphate buffer (pH 8.0). Data were collected from 260 nm to 190 nm at 25 °C in a cuvette with a 1-mm path length. The sensitivity was set at 100 mdeg, and the scanning speed was 100 nm/min. For each batch of monomers, the spectrum reported is the sum of three separate scans. The secondary structures of the monomers were estimated from their CD spectra with the CDSSTR algorithm [55,56,57] using the DichroWeb server [58,59,60] and reference dataset SET7 (soluble globular and denatured proteins).

### 4.11. Seed Amplification Assay (SAA)

The SAA was performed as described in our prior publications [14,15,16]. A 96-well clear-bottom plate (Thermo Fisher Scientific, Waltham, MA, USA; 1256672) was loaded with six 800-µm silica beads (OPS Diagnostics, Lebanon, NJ, USA; 80020002) in each well. Purified αSyn monomer was thawed from −80 °C and centrifuge-filtered (100 kDa) to remove aggregates that may have formed in storage. The reaction mixture contained 0.1 mg/mL filtered αSyn monomer (as measured by the NanoDrop), 10 µM ThT, 40 mM phosphate buffer (pH 8.0), 0.00125% SDS, and varying concentrations of NaCl depending on the experimentally determined optimized concentration. Next, 98 µL of the reaction mixture was loaded into each well of the plate followed by 2 µL of biomatrix from the specified dilution. The plates were sealed with a plate sealer (Thermo Fisher Scientific, Waltham, MA, USA; 235307) and incubated in the CLARIOstar microplate reader (BMG Labtech, Ortenberg, Germany) at 42 °C. The reader settings were as follows: double orbital shaking at 400 rpm for 1 min followed by 1-min resting incubation between shaking cycles; the gain setting was between 1600 and 1800; ThT fluorescence was measured every 30 min for a minimum of 42 h using 445-nm excitation and 480-nm emission wavelengths. The samples were run as quadruplicates unless otherwise specified. The kinetic parameters evaluated were protein aggregation rate (PAR), fluorescence maximum (Fmax), hit rate, and fidelity, which are defined in Table S1. The threshold line used to calculate PAR and hit rate is defined as the average of the first ten readings of all samples plus ten standard deviations (SDs). The algorithm used to predict PD patients vs. controls in the blinded skin biopsy study is described in the Supplementary Materials.

### 4.12. Ionic Strength Optimization

To optimize ionic strength in the SAA, we used both a DLB brain homogenate and a healthy control brain homogenate to seed the SAA reaction in each new batch of αSyn monomers. The objective of our optimization was to enhance the Fmax of the DLB sample (Fmax_DLB_) while minimizing the Fmax of the healthy control (Fmax_con_). The metric used here was fidelity (Fmax_DLB_/Fmax_con_) to reflect the fold difference in maximum seeding activity of the disease vs. control. This is similar but distinct from a metric used by Metrick et al., 2019 who used fidelity to describe the fold difference in lag time [30]. NaCl concentrations were adjusted from 0 to 170 mM, while the concentrations of all other reaction mixture components were held constant (e.g., 0.1 mg/mL monomer, 40 mM phosphate buffer, 0.00125% SDS, 10 µM ThT). The NaCl concentration with the largest fidelity was selected as the optimal salt concentration for the given batch. Following ionic strength optimization, the kinetic variability between batches was measured and detailed in the “Statistics” section.

### 4.13. Comparative Analysis of In-House Monomer vs. Proprietary Monomer

Our WT in-house monomer was compared against two proprietary αSyn monomers that have been used in other publications [26,49]. The Proteos WT monomer was compared to three batches of our in-house monomer using PD and control brain homogenates in our SAA conditions. The K23Q αSyn monomer from Impact Biologicals Inc. (Swarthmore, PA, USA; 31101) was compared to one of our in-house batches using neuropathologically confirmed PD (*n* = 4) and healthy control (*n* = 1) skin autopsy samples with our SAA conditions.

### 4.14. External Lab Validation of Monomer Efficacy in SAA

The SAA assay described above was further validated independently by a second laboratory with changes to the reaction mixture and plate reader settings. In brief, the SAA reaction mix was composed of 40 mM phosphate buffer at pH 8.4, 40 mM NaCl, 0.0013% SDS, and 10 µM ThT, and this mixture was filtered through a 0.22-µm filter before use. The αSyn monomer was filtered with a 100-kDa spin column filter (Millipore) and then gently added to the SAA reaction mix. Then, 98 µL of SAA reaction mix containing 10 µg αSyn was loaded into each well of a 96-well plate (Nunc, Thermo Fisher Scientific, Waltham, MA, USA; 165305). Postmortem brain and skin homogenates from neuropathologically confirmed PD and non-PD cases were used to seed the reaction. The 10% tissue homogenates were diluted at 1:100 and 1:1000 in 1× PBS containing 1 × N2 supplement (Gibco, Thermo Fisher Scientific Waltham, MA, USA; 17502048) and centrifuged at 5000× *g* for 5 min at 4 °C. Next, 2 μL of diluted homogenate was added to the 98 μL of RT-QuIC reaction mix in each well with six 800-μm glass beads (OPS Diagnostics, Lebanon, NJ, USA). The plate was then sealed with plate sealing tape (Nunc, Thermo Fisher Scientific, MA, USA) and incubated at 42 °C in a BMG FLUOstar Omega plate reader (BMG Labtech, Ortenberg, Germany) with cycles of 1-min shaking (400-rpm double orbital) and 29-min rest throughout the incubation time. The ThT fluorescence measurements were monitored using excitation and emission wavelengths of 450 ± 10 nm and 480 ± 10 nm, respectively, by bottom reading.

### 4.15. Statistics

Each resulting SAA kinetic curve was fitted using an asymmetric 5-parameter logistic function (Equation (1)) using the MARS software V4.20 (BMG Labtech, Ortenberg, Germany) to account for the noise in fluorescence readings and consistently quantify the kinetic metrics. The 5-parameter fit function was chosen based on the asymmetric nature of SAA kinetic curves. Thus, incorporating the symmetry of the curve increased the goodness-of-fit of the model to the experimental data [61]. Moreover, this equation is similar to the empirical work completed by Fink et al. [62] describing a mathematical model of amyloid fibril formation. The output variables from the function were used to derive the Fmax, PAR, fidelity, and hit rate. The Supplementary Materials give the specifics of how the Fmax, PAR, fidelity, and hit rate were derived from the 5-parameter fit model.

(1) $$y=Bottom+\frac{Top-Bottom}{{\left[1+{\left(\frac{IP}{x}\right)}^{Slope}\right]}^{Sym}}$$

In this equation, the parameters *Bottom*, *Top*, *IP*, *x*, *Slope*, and *Sym* correspond to the ground asymptote, maximum asymptote, inflection point, x-axis (time in hours), slope, and degree of symmetry, respectively. The algebraic manipulation of the function to obtain the PAR is shown in Equation (S1) in the Supplementary Materials. The SD and CV were used to quantify the repeatability of the resulting kinetic metrics in absolute and relative terms, respectively. Here, we have deliberately used the terms precision, repeatability, reproducibility, and robustness to follow the nomenclature provided by [10] and the suggested data for each type of validation [53], as clearly defined in Table S2. GraphPad 8.0 or 9.2 was used to plot and analyze raw data. The MARS software V4.20 (BMG Labtech, Ortenberg, Germany) was used to plot the fitted replicate curves. An example of how this was carried out is shown in Figure S2. Student’s *t*-test was used to compare the two groups, which were considered statistically significant for *p*-values ≤ 0.05.

## Figures and Tables

**Table 1:** Average yield of αSyn purification using our protein purification protocol.

Monomer Batch	Total α-Syn Yield
αSyn Batch 1	76 mg
αSyn Batch 2	65 mg
αSyn Batch 3	84 mg
αSyn Batch 4	68 mg
αSyn Batch 5	45 mg
αSyn Batch 6	62 mg
αSyn Batch 7	81 mg
Average Yield ± SD	68.65 ± 12.26 mg

## Data Availability

The fully anonymized datasets used and analyzed during the current study are available from the corresponding authors upon reasonable request.

## Supplementary Materials

The following supporting information can be downloaded at: https://www.mdpi.com/article/10.3390/ijms25115988/s1.

## Abbreviations

αSyn: Alpha-synuclein; SAAs: seed amplification assays; PD: Parkinson’s disease; SMG: submandibular gland; FPLC: fast-protein liquid chromatography; Fmax: fluorescence maximum; ICH: International Council for Harmonization; ThT: Thioflavin T; E. coli: Escherichia coli; DLB: dementia with Lewy bodies; RT: room temperature; SEC: size-exclusion chromatography; CV: column-volumes; DI: deionized; MALDI-TOF: matrix-assisted laser desorption ionization time-of-flight; PFFs: preformed fibrils; MJFF: Michael J. Fox Foundation; TEM: transmission electron microscopy; CD: circular dichroism; PAR: protein aggregation rate; SD: standard deviation; RFU: relative fluorescence units.

## References

[1] Marti M.J., Tolosa E., Campdelacreu J. (2003). Clinical overview of the synucleinopathies. Mov. Disord..

[2] Siderowf A., Concha-Marambio L., Lafontant D.E., Farris C.M., Ma Y., Urenia P.A., Nguyen H., Alcalay R.N., Chahine L.M., Foroud T. (2023). Assessment of heterogeneity among participants in the Parkinson’s Progression Markers Initiative cohort using alpha-synuclein seed amplification: A cross-sectional study. Lancet Neurol..

[3] Kang U.J., Boehme A.K., Fairfoul G., Shahnawaz M., Ma T.C., Hutten S.J., Green A., Soto C. (2019). Comparative study of cerebrospinal fluid alpha-synuclein seeding aggregation assays for diagnosis of Parkinson’s disease. Mov. Disord..

[4] Donadio V., Incensi A., Del Sorbo F., Rizzo G., Infante R., Scaglione C., Modugno N., Fileccia E., Elia A.E., Cencini F. (2018). Skin Nerve Phosphorylated alpha-Synuclein Deposits in Parkinson Disease with Orthostatic Hypotension. J. Neuropathol. Exp. Neurol..

[5] Donadio V., Incensi A., Leta V., Giannoccaro M.P., Scaglione C., Martinelli P., Capellari S., Avoni P., Baruzzi A., Liguori R. (2014). Skin nerve alpha-synuclein deposits: A biomarker for idiopathic Parkinson disease. Neurology.

[6] Ikemura M., Saito Y., Sengoku R., Sakiyama Y., Hatsuta H., Kanemaru K., Sawabe M., Arai T., Ito G., Iwatsubo T. (2008). Lewy body pathology involves cutaneous nerves. J. Neuropathol. Exp. Neurol..

[7] Beach T.G., Adler C.H., Sue L.I., Vedders L., Lue L., White Iii C.L., Akiyama H., Caviness J.N., Shill H.A., Sabbagh M.N. (2010). Multi-organ distribution of phosphorylated alpha-synuclein histopathology in subjects with Lewy body disorders. Acta Neuropathol..

[8] Braak H., de Vos R.A., Bohl J., Del Tredici K. (2006). Gastric alpha-synuclein immunoreactive inclusions in Meissner’s and Auerbach’s plexuses in cases staged for Parkinson’s disease-related brain pathology. Neurosci. Lett..

[9] Wang N., Gibbons C.H., Lafo J., Freeman R. (2013). alpha-Synuclein in cutaneous autonomic nerves. Neurology.

[10] Adler C.H., Dugger B.N., Hinni M.L., Lott D.G., Driver-Dunckley E., Hidalgo J., Henry-Watson J., Serrano G., Sue L.I., Nagel T. (2014). Submandibular gland needle biopsy for the diagnosis of Parkinson disease. Neurology.

[11] Beach T.G., Adler C.H., Serrano G., Sue L.I., Walker D.G., Dugger B.N., Shill H.A., Driver-Dunckley E., Caviness J.N., Intorcia A. (2016). Prevalence of Submandibular Gland Synucleinopathy in Parkinson’s Disease, Dementia with Lewy Bodies and other Lewy Body Disorders. J. Parkinsons Dis..

[12] Okuzumi A., Hatano T., Matsumoto G., Nojiri S., Ueno S.I., Imamichi-Tatano Y., Kimura H., Kakuta S., Kondo A., Fukuhara T. (2023). Propagative alpha-synuclein seeds as serum biomarkers for synucleinopathies. Nat. Med..

[13] Shannon K.M., Keshavarzian A., Mutlu E., Dodiya H.B., Daian D., Jaglin J.A., Kordower J.H. (2012). Alpha-synuclein in colonic submucosa in early untreated Parkinson’s disease. Mov. Disord..

[14] Manne S., Kondru N., Jin H., Serrano G.E., Anantharam V., Kanthasamy A., Adler C.H., Beach T.G., Kanthasamy A.G. (2020). Blinded RT-QuIC Analysis of alpha-Synuclein Biomarker in Skin Tissue From Parkinson’s Disease Patients. Mov. Disord..

[15] Manne S., Kondru N., Jin H., Anantharam V., Huang X., Kanthasamy A., Kanthasamy A.G. (2020). alpha-Synuclein real-time quaking-induced conversion in the submandibular glands of Parkinson’s disease patients. Mov. Disord..

[16] Manne S., Kondru N., Hepker M., Jin H., Anantharam V., Lewis M., Huang X., Kanthasamy A., Kanthasamy A.G. (2019). Ultrasensitive Detection of Aggregated alpha-Synuclein in Glial Cells, Human Cerebrospinal Fluid, and Brain Tissue Using the RT-QuIC Assay: New High-Throughput Neuroimmune Biomarker Assay for Parkinsonian Disorders. J. Neuroimmune Pharmacol..

[17] Bargar C., Wang W., Gunzler S.A., LeFevre A., Wang Z., Lerner A.J., Singh N., Tatsuoka C., Appleby B., Zhu X. (2021). Streamlined alpha-synuclein RT-QuIC assay for various biospecimens in Parkinson’s disease and dementia with Lewy bodies. Acta Neuropathol. Commun..

[18] Mammana A., Baiardi S., Quadalti C., Rossi M., Donadio V., Capellari S., Liguori R., Parchi P. (2021). RT-QuIC Detection of Pathological alpha-Synuclein in Skin Punches of Patients with Lewy Body Disease. Mov. Disord..

[19] Kuzkina A., Bargar C., Schmitt D., Rossle J., Wang W., Schubert A.L., Tatsuoka C., Gunzler S.A., Zou W.Q., Volkmann J. (2021). Diagnostic value of skin RT-QuIC in Parkinson’s disease: A two-laboratory study. NPJ Park. Dis..

[20] Iranzo A., Mammana A., Munoz-Lopetegi A., Dellavalle S., Maya G., Rossi M., Serradell M., Baiardi S., Arqueros A., Quadalti C. (2023). Misfolded alpha-Synuclein Assessment in the Skin and CSF by RT-QuIC in Isolated REM Sleep Behavior Disorder. Neurology.

[21] Fairfoul G., McGuire L.I., Pal S., Ironside J.W., Neumann J., Christie S., Joachim C., Esiri M., Evetts S.G., Rolinski M. (2016). Alpha-synuclein RT-QuIC in the CSF of patients with alpha-synucleinopathies. Ann. Clin. Transl. Neurol..

[22] Concha-Marambio L., Pritzkow S., Shahnawaz M., Farris C.M., Soto C. (2023). Seed amplification assay for the detection of pathologic alpha-synuclein aggregates in cerebrospinal fluid. Nat. Protoc..

[23] Paciotti S., Bellomo G., Gatticchi L., Parnetti L. (2018). Are We Ready for Detecting alpha-Synuclein Prone to Aggregation in Patients? The Case of “Protein-Misfolding Cyclic Amplification” and “Real-Time Quaking-Induced Conversion” as Diagnostic Tools. Front. Neurol..

[24] Jucker M., Walker L.C. (2013). Self-propagation of pathogenic protein aggregates in neurodegenerative diseases. Nature.

[25] Russo M.J., Orru C.D., Concha-Marambio L., Giaisi S., Groveman B.R., Farris C.M., Holguin B., Hughson A.G., LaFontant D.E., Caspell-Garcia C. (2021). High diagnostic performance of independent alpha-synuclein seed amplification assays for detection of early Parkinson’s disease. Acta Neuropathol. Commun..

[26] Martinez-Valbuena I., Visanji N.P., Kim A., Lau H.H.C., So R.W.L., Alshimemeri S., Gao A., Seidman M.A., Luquin M.R., Watts J.C. (2022). Alpha-synuclein seeding shows a wide heterogeneity in multiple system atrophy. Transl. Neurodegener..

[27] Greenfield N., Fasman G.D. (1969). Computed circular dichroism spectra for the evaluation of protein conformation. Biochemistry.

[28] Groveman B.R., Orru C.D., Hughson A.G., Raymond L.D., Zanusso G., Ghetti B., Campbell K.J., Safar J., Galasko D., Caughey B. (2018). Rapid and ultra-sensitive quantitation of disease-associated alpha-synuclein seeds in brain and cerebrospinal fluid by alphaSyn RT-QuIC. Acta Neuropathol. Commun..

[29] Concha-Marambio L., Farris C.M., Holguin B., Ma Y., Seibyl J., Russo M.J., Kang U.J., Hutten S.J., Merchant K., Shahnawaz M. (2021). Seed Amplification Assay to Diagnose Early Parkinson’s and Predict Dopaminergic Deficit Progression. Mov. Disord..

[30] Metrick M.A., do Carmo Ferreira N., Saijo E., Hughson A.G., Kraus A., Orru C., Miller M.W., Zanusso G., Ghetti B., Vendruscolo M. (2019). Million-fold sensitivity enhancement in proteopathic seed amplification assays for biospecimens by Hofmeister ion comparisons. Proc. Natl. Acad. Sci. USA.

[31] Hoyer W., Antony T., Cherny D., Heim G., Jovin T.M., Subramaniam V. (2002). Dependence of alpha-synuclein aggregate morphology on solution conditions. J. Mol. Biol..

[32] Buell A.K., Galvagnion C., Gaspar R., Sparr E., Vendruscolo M., Knowles T.P., Linse S., Dobson C.M. (2014). Solution conditions determine the relative importance of nucleation and growth processes in alpha-synuclein aggregation. Proc. Natl. Acad. Sci. USA.

[33] Wang Z., Becker K., Donadio V., Siedlak S., Yuan J., Rezaee M., Incensi A., Kuzkina A., Orru C.D., Tatsuoka C. (2020). Skin alpha-Synuclein Aggregation Seeding Activity as a Novel Biomarker for Parkinson Disease. JAMA Neurol..

[34] Islam M.S., Aryasomayajula A., Selvaganapathy P.S. (2017). A Review on Macroscale and Microscale Cell Lysis Methods. Micromachines.

[35] Fonseca L.P., Cabral J.M.S. (2002). Penicillin acylase release from *Escherichia coli* cells by mechanical cell disruption and permeabilization. J. Chem. Technol. Biotechnol..

[36] Stephens A.D., Matak-Vinkovic D., Fernandez-Villegas A., Kaminski Schierle G.S. (2020). Purification of Recombinant alpha-synuclein: A Comparison of Commonly Used Protocols. Biochemistry.

[37] Huang C., Ren G., Zhou H., Wang C.C. (2005). A new method for purification of recombinant human alpha-synuclein in Escherichia coli. Protein Expr. Purif..

[38] Ren G., Wang X., Hao S., Hu H., Wang C.C. (2007). Translocation of alpha-synuclein expressed in Escherichia coli. J. Bacteriol..

[39] Morris A.M., Finke R.G. (2009). Alpha-synuclein aggregation variable temperature and variable pH kinetic data: A re-analysis using the Finke-Watzky 2-step model of nucleation and autocatalytic growth. Biophys. Chem..

[40] Candelise N., Schmitz M., Thune K., Cramm M., Rabano A., Zafar S., Stoops E., Vanderstichele H., Villar-Pique A., Llorens F. (2020). Effect of the micro-environment on alpha-synuclein conversion and implication in seeded conversion assays. Transl. Neurodegener..

[41] Coelho-Cerqueira E., Carmo-Goncalves P., Pinheiro A.S., Cortines J., Follmer C. (2013). alpha-Synuclein as an intrinsically disordered monomer—Fact or artefact?. FEBS J..

[42] Roeters S.J., Iyer A., Pletikapic G., Kogan V., Subramaniam V., Woutersen S. (2017). Evidence for Intramolecular Antiparallel Beta-Sheet Structure in Alpha-Synuclein Fibrils from a Combination of Two-Dimensional Infrared Spectroscopy and Atomic Force Microscopy. Sci. Rep..

[43] Tartaglia G.G., Pawar A.P., Campioni S., Dobson C.M., Chiti F., Vendruscolo M. (2008). Prediction of aggregation-prone regions in structured proteins. J. Mol. Biol..

[44] Bousset L., Pieri L., Ruiz-Arlandis G., Gath J., Jensen P.H., Habenstein B., Madiona K., Olieric V., Bockmann A., Meier B.H. (2013). Structural and functional characterization of two alpha-synuclein strains. Nat. Commun..

[45] Bellomo G., Paciotti S., Gatticchi L., Rizzo D., Paoletti F.P., Fragai M., Parnetti L. (2021). Seed amplification assays for diagnosing synucleinopathies: The issue of influencing factors. Front. Biosci..

[46] Madelin G., Regatte R.R. (2013). Biomedical applications of sodium MRI in vivo. J. Magn. Reson. Imaging.

[47] Grimaldi S., El Mendili M.M., Zaaraoui W., Ranjeva J.P., Azulay J.P., Eusebio A., Guye M. (2021). Increased Sodium Concentration in Substantia Nigra in Early Parkinson’s Disease: A Preliminary Study With Ultra-High Field (7T) MRI. Front. Neurol..

[48] Mellon E.A., Pilkinton D.T., Clark C.M., Elliott M.A., Witschey W.R., Borthakur A., Reddy R. (2009). Sodium MR imaging detection of mild Alzheimer disease: Preliminary study. AJNR Am. J. Neuroradiol..

[49] Jin Y., Li F., Sonoustoun B., Kondru N.C., Martens Y.A., Qiao W., Heckman M.G., Ikezu T.C., Li Z., Burgess J.D. (2022). APOE4 exacerbates alpha-synuclein seeding activity and contributes to neurotoxicity in Alzheimer’s disease with Lewy body pathology. Acta Neuropathol..

[50] Koo H.J., Lee H.J., Im H. (2008). Sequence determinants regulating fibrillation of human alpha-synuclein. Biochem. Biophys. Res. Commun..

[51] Coskuner O., Wise-Scira O. (2013). Structures and free energy landscapes of the A53T mutant-type alpha-synuclein protein and impact of A53T mutation on the structures of the wild-type alpha-synuclein protein with dynamics. ACS Chem. Neurosci..

[52] Virameteekul S., Revesz T., Jaunmuktane Z., Warner T.T., De Pablo-Fernandez E. (2023). Clinical Diagnostic Accuracy of Parkinson’s Disease: Where Do We Stand?. Mov. Disord..

[53] U (2024). S Food & Drug Administration. Q2(R2) Validation of Analytical Procedures.

[54] Becker K., Wang X., Vander Stel K., Chu Y., Kordower J., Ma J. (2018). Detecting Alpha Synuclein Seeding Activity in Formaldehyde-Fixed MSA Patient Tissue by PMCA. Mol. Neurobiol..

[55] Sreerama N., Woody R.W. (2000). Estimation of protein secondary structure from circular dichroism spectra: Comparison of CONTIN, SELCON, and CDSSTR methods with an expanded reference set. Anal Biochem..

[56] Sreerama N., Venyaminov S.Y., Woody R.W. (2000). Estimation of protein secondary structure from circular dichroism spectra: Inclusion of denatured proteins with native proteins in the analysis. Anal Biochem..

[57] Compton L.A., Johnson W.C. (1986). Analysis of protein circular dichroism spectra for secondary structure using a simple matrix multiplication. Anal Biochem..

[58] Miles A.J., Ramalli S.G., Wallace B.A. (2022). DichroWeb, a website for calculating protein secondary structure from circular dichroism spectroscopic data. Protein Sci..

[59] Whitmore L., Wallace B.A. (2008). Protein secondary structure analyses from circular dichroism spectroscopy: Methods and reference databases. Biopolymers.

[60] Whitmore L., Wallace B.A. (2004). DICHROWEB, an online server for protein secondary structure analyses from circular dichroism spectroscopic data. Nucleic Acids Res..

[61] Spiess A.N., Feig C., Ritz C. (2008). Highly accurate sigmoidal fitting of real-time PCR data by introducing a parameter for asymmetry. BMC Bioinform..

[62] Nielsen L., Khurana R., Coats A., Frokjaer S., Brange J., Vyas S., Uversky V.N., Fink A.L. (2001). Effect of environmental factors on the kinetics of insulin fibril formation: Elucidation of the molecular mechanism. Biochemistry.

