# Supplementary material for: High-Yield α-Synuclein Purification and Ionic Strength Modification Pivotal to Seed Amplification Assay Performance and Reproducibility
Source: Int J Mol Sci. 2024 May 30;25(11):5988. doi: 10.3390/ijms25115988 (PMC11172462; doi:10.3390/ijms25115988)
Supplement: Supplementary file 1 [file ijms-25-05988-s001.zip › ijms-2992340-supplementary.pdf]

Supplementary Materials

The protein aggregation rate (PAR) is the time it takes for the replicate to cross the threshold line. The curve was fitted to the 5-parameter fit function shown in Equation 1 using the MARS software. The threshold line was calculated by taking the average of all bottom asymptote readouts (shown in the equation as "Bottom") plus 10 times the standard deviation (SD) of all "Bottom" readouts. If the fit function did not yield a "Bottom" parameter, it was estimated by taking the average of the first ten readings of that replicate. The parameters Bottom, Top, IP, Slope, and Sym correspond to the ground asymptote, maximum asymptote, inflection point, slope, and degree of symmetry, respectively. Once the output variables were obtained, the function was set to X and the threshold line fluorescence was given as Y to yield an equation for Time to Threshold (TtT). The algebraic manipulation of Equation 1 to obtain TtT is shown in Equation S1. Once the TtT was obtained, the PAR was calculated with 1/TtT. If the replicate did not cross threshold, it was given a PAR of 0.024 (1/42 h).

$$TtT = \frac{IP}{\sqrt[Slope]{\sqrt[Sym]{\frac{Top - Bottom}{Y - Bottom}} - 1}}$$

(Equation S1)

The algorithm used to predict positivity of the skin biopsy study was the following:

1. For every replicate with a PAR $\geq$ 0.042 (crosses threshold within 24 h), the sample was given 1 point.
2. If the average Fmax intensity across all replicates is greater than (Threshold Fluorescence * 1.5), the sample was given 1 point.
3. If the sample had a final score of at least 3, it was considered positive while scores of 2 or less were considered negative.

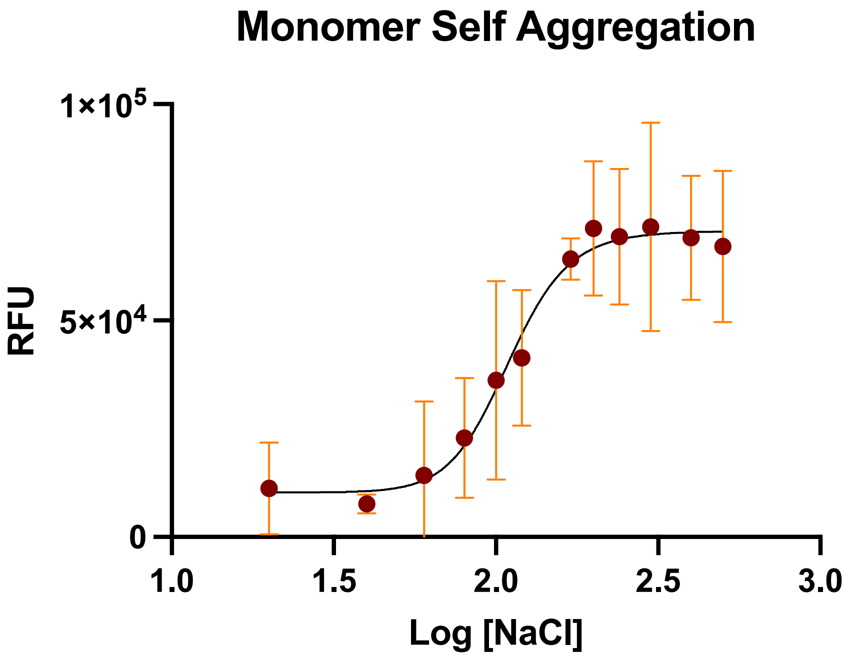

Figure S1. Self aggregation of αSyn monomer plateaus after 170 mM NaCl. Fmax ThT fluorescence of αSyn monomer with reaction buffers containing NaCl concentrations ranging from 0-500 mM.

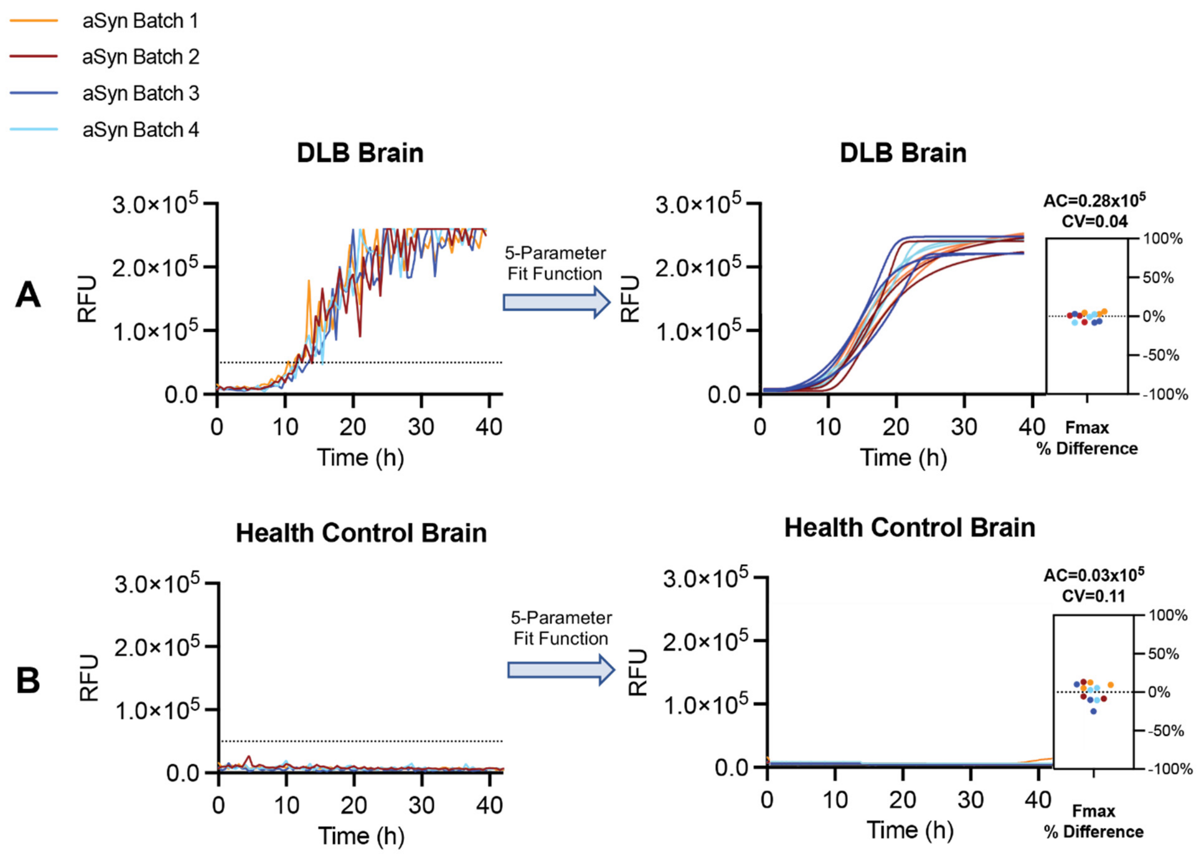

Figure S2. Raw data conversion to 5-parameter fit for kinetic curve analysis. The raw fluorescence was averaged and plotted in Figure 4 using GraphPad 8.0. For kinetic curve analysis, the raw data of each replicate was fitted to a 5-parameter fit function in MARS (V4.20) and output variables were used for the dot plots of DLB brain (A) and healthy control brain (B). In this case, the Fmax at 40 hours was used to plot the Fmax % difference.

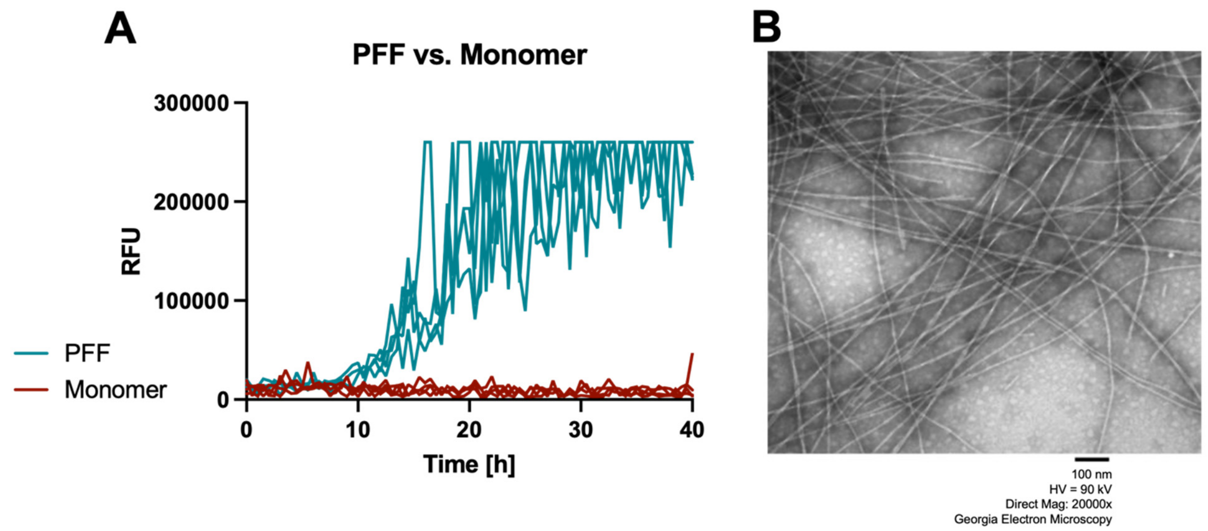

Figure S3. Pre-formed fibril seed αSyn SAA. (A) SAA using PFF to seed the reaction or monomer alone. (B) TEM of PFF generated using the MJFF protocol from the same monomer shown in Figure 2E.

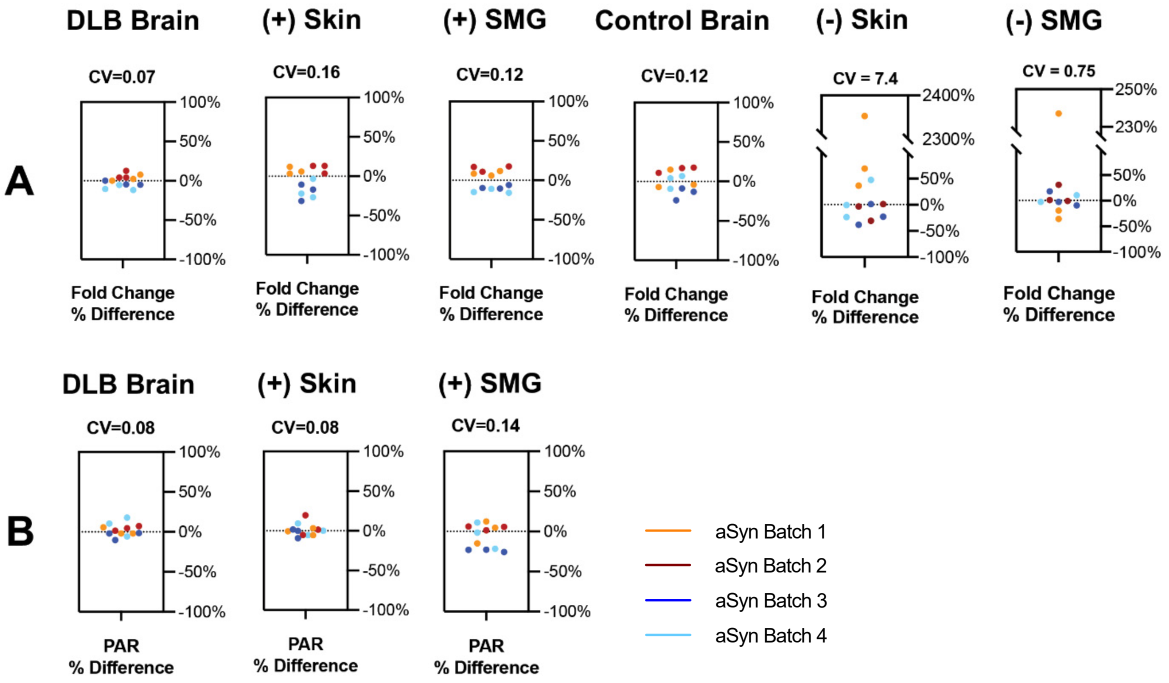

Figure S4. Dot plots of fold difference and PAR percent difference from median. The percent difference of each replicate from the median was computed for the Fmax fold difference (A) and PAR (B) from each batch of αSyn monomer. Each dot represents one replicate color-coded by batch of αSyn monomer.

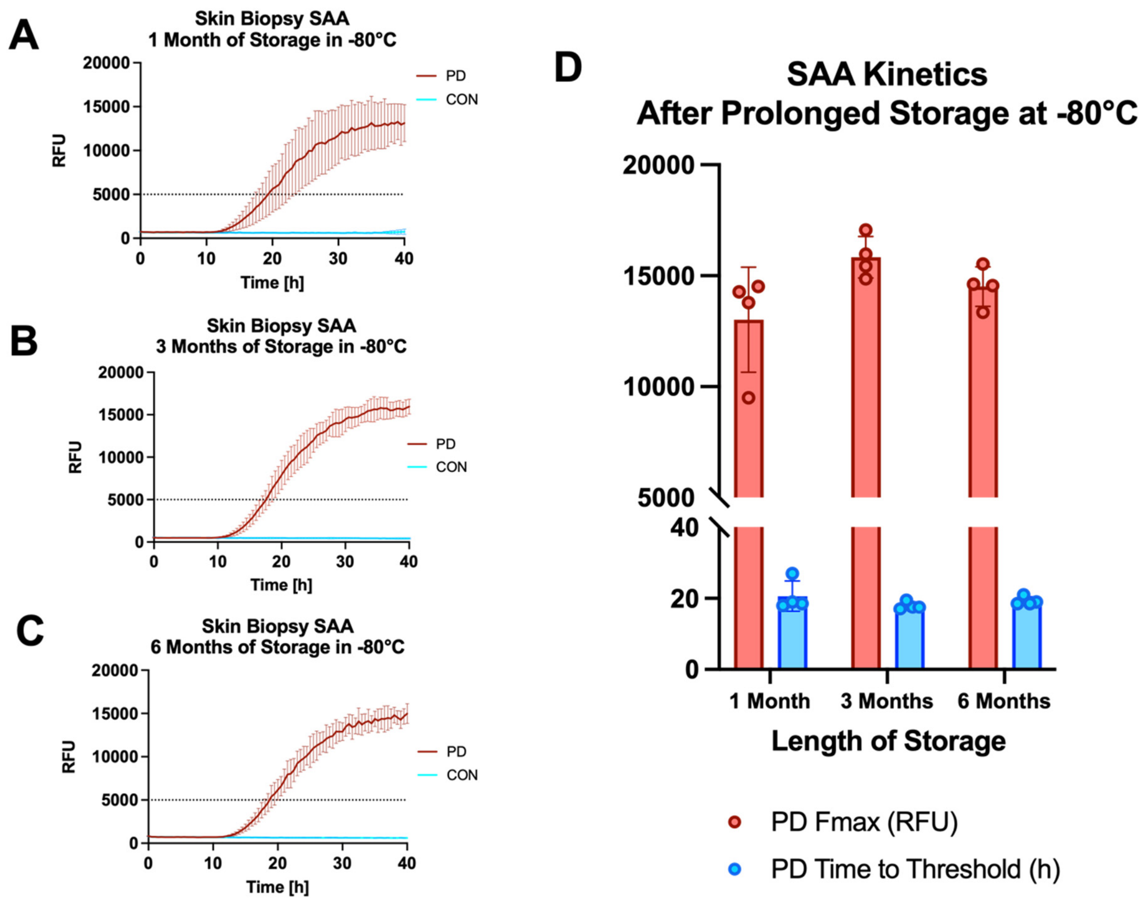

Figure S5. Our in-house αSyn monomer is stable over long periods of storage at -80°C. SAA using our in-house monomer seeded with skin biopsy samples from PD (*n*=1) and control (*n*=1) at 1 month (A), 3 months (B), or 6 months (C) of storage at -80°C. Aggregation kinetics of the PD sample is shown at each time point (D).

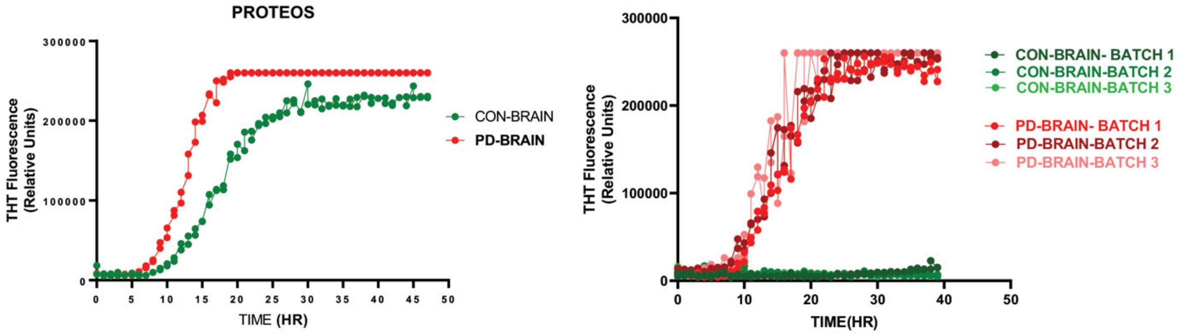

Figure S6. Comparison of in-house and commercial αSyn monomer in SAA. Proteos commercial monomer showing false-positive seeding activity among the control brain homogenate batches compared to the batch-to-batch consistency of our in-house lab monomer when it comes to differentiating true positive and true negative seeding activity between PD brain and control brain homogenates in αSyn seed amplification assays.

Table S1. Definitions of aggregation kinetic parameters.

Parameter	Definition	Calculation	Use Case
**Protein Aggregation Rate (PAR)**	Rate of kinetic curve crossing the threshold line	$\frac{1}{TtT}$	Calculate Positivity
**Fluorescence Maximum (Fmax)**	The maximum RFU value (Top asymptote of 5-P fit)	Top at 48 h (or last 10 time points)	Calculate Positivity
**Hit Rate**	Number of Replicates crossing threshold line before 24 hours	$\frac{Reps.\,Crossing\ Threshold\ before\ 24h}{Total\ Reps.}$	Calculate Positivity
**Fidelity**	Fold difference between the Fmax of the positive sample compared to the Fmax of healthy control	$\frac{Fmax_{pos}}{Fmax_{con}}$	Optimize Reaction Conditions

Table S2. Definitions of ICH nomenclature and recommended data for validation of analytical procedures.

	Definition	Recommended Data
**Precision**	Closeness of agreement between a series of measurements obtained from multiple samplings of the same homogenous sample under the prescribed conditions. Precision is considered at three levels: repeatability, intermediate precision, and reproducibility.	Usually expressed as the variance, standard deviation, or coefficient of variation of a series of measurements using homogenous, authentic samples or artificially prepared samples.
**Repeatability**	Expressed the precision under the same operating conditions over a short interval of time. Also termed intra-assay precision.	Technical replicate variability.
**Intermediate Precision**	Expresses within-laboratory variations. Factors to be considered should include potential sources of variability, e.g., different days, different environmental conditions, different analysts, and different equipment.	Inter-batch variation, different days, different environmental conditions, and different analysts.
**Reproducibility**	Expresses the precision between laboratories.	Inter-laboratory studies.
**Robustness**	Measure of its capacity to meet the expected performance requirements during normal use.	Deliberate variations of analytical procedure parameters.

Table S3. Predicted secondary structural elements of αSyn samples from circular dichroism spectra.

Sample	α-Helix (%)	b-Strand (%)	b-Turn (%)	Unordered (%)
**Batch 1**	2	11	7	79
**Batch 2**	2	10	7	80
**Batch 3**	2	9	7	81
**Batch 4**	0	17	11	68
**Batch 5**	1	13	7	78
**Batch 6**	1	8	5	84
**Batch 7**	1	7	5	86

Table S4. Average of SAA kinetic parameters of four protein batches using brain, skin, and SMG.

		DLB Brain	(+) Skin	(+) SMG	(–) Brain	(–) Skin	(–) SMG
**Batch 1**	Average Fmax	262202	234506	259069	8311	3717	10260
	Average PAR	0.082	0.072	0.075	N/A	N/A	N/A
	Fold Change	48.11	49.70	48.21	1.46	0.65	1.81
	Hit Rate	3/3	3/3	3/3	0/3	0/3	0/3
**Batch 2**	Average Fmax	254188	241487	251048	7605	4137	7060
	Average PAR	0.0789	0.0674	0.0720	N/A	N/A	N/A
	Fold Change	46.45	48.37	45.54	1.34	0.73	1.24
	Hit Rate	3/3	3/3	3/3	0/3	0/3	0/3
**Batch 3**	Average Fmax	248370	198268	215609	6368	5007	6525
	Average PAR	0.0748	0.0663	0.0545	N/A	N/A	N/A
	Fold Change	43.49	36.31	38.26	1.12	0.88	1.15
	Hit Rate	3/3	3/3	3/3	0/3	0/3	0/3
**Batch 4**	Average Fmax	241152	222639	244664	7561	46540	8963
	Average PAR	0.0843	0.0690	0.0687	N/A	N/A	N/A
	Fold Change	40.83	37.38	36.11	1.33	8.20	1.58
	Hit Rate	3/3	3/3	3/3	0/3	1/3	1/3
